# Comparative Transcriptomics of Non-Embryogenic and Embryogenic Callus in Semi-Recalcitrant and Non-Recalcitrant Upland Cotton Lines

**DOI:** 10.3390/plants10091775

**Published:** 2021-08-26

**Authors:** Sonika Kumar, Ashleigh Ruggles, Sam Logan, Alora Mazarakis, Thomas Tyson, Matthew Bates, Clayton Grosse, David Reed, Zhigang Li, Jane Grimwood, Jeremy Schmutz, Christopher Saski

**Affiliations:** 1 Department of Plant and Environmental Sciences, Clemson University, Clemson, SC 29634, USA; sonikak@clemson.edu (S.K.); zhiganl@clemson.edu (Z.L.); 2 Techshot Inc., Greenville, IN 47124, USA; aruggles@techshot.com (A.R.); slogan@techshot.com (S.L.); amazarakis@techshot.com (A.M.); ttyson@techshot.com (T.T.); mbates@techshot.com (M.B.); cgrosse@techshot.com (C.G.); dreed@techshot.com (D.R.); 3 HudsonAlpha Institute for Biotechnology, Huntsville, AL 35806, USA; jgrimwood@hudsonalpha.org (J.G.); jschmutz@hudsonalpha.org (J.S.)

**Keywords:** genotype-specific recalcitrance, reprogramming, somatic embryogenesis, *Gossypium hirsutum* L.

## Abstract

Somatic embryogenesis-mediated plant regeneration is essential for the genetic manipulation of agronomically important traits in upland cotton. Genotype specific recalcitrance to regeneration is a primary challenge in deploying genome editing and incorporating useful transgenes into elite cotton germplasm. In this study, transcriptomes of a semi-recalcitrant cotton (*Gossypium hirsutum* L.) genotype ‘Coker312’ were analyzed at two critical stages of somatic embryogenesis that include non-embryogenic callus (NEC) and embryogenic callus (EC) cells, and the results were compared to a non-recalcitrant genotype ‘Jin668’. We discovered 305 differentially expressed genes in Coker312, whereas, in Jin668, about 6-fold more genes (2155) were differentially expressed. A total of 154 differentially expressed genes were common between the two genotypes. Gene enrichment analysis of the upregulated genes identified functional categories, such as lipid transport, embryo development, regulation of transcription, sugar transport, and vitamin biosynthesis, among others. In Coker312 EC cells, five major transcription factors were highly upregulated: *LEAFY COTYLEDON 1 (LEC1)*, *WUS-related homeobox 5 (WOX5)*, *ABSCISIC ACID INSENSITIVE3 (ABI3)*, *FUSCA3 (FUS3)*, and *WRKY2*. In Jin668, *LEC1*, *BABY BOOM (BBM)*, *FUS3*, and *AGAMOUS-LIKE15 (AGL15)* were highly expressed in EC cells. We also found that gene expression of these embryogenesis genes was typically higher in Jin668 when compared to Coker312. We conclude that significant differences in the expression of the above genes between Coker312 and Jin668 may be a critical factor affecting the regenerative ability of these genotypes.

## 1. Introduction

Plant somatic embryogenesis (SE) is a unique developmental process that ultimately leads to the regeneration of a whole plant from single or multiple somatic cells [1]. This process involves sophisticated cellular reprogramming events that are controlled by gene expression programs and signaling pathways that direct callus cells to dedifferentiate, reprogram, and begin differentiation into polarized structures that eventually become a viable embryo [2,3,4,5]. SE is initiated by various factors, such as culture medium conditions, including concentrations, plant growth regulators (PGRs), and various stresses, such as plant wounding, temperature, and osmotic pressures [3,6,7]. Under SE initiation, somatic cells from various explant sources (e.g., hypocotyls, young leaves, and immature embryos) form non-embryogenic callus cells (NEC) that can be described as unorganized, dedifferentiated, continuously dividing cell masses. These cells are responsive to the components in the growth medium and signals from the environment, such as light and temperature. Eventually, cells with embryogenic potential will differentiate into embryogenic callus cells (EC), which are cells that are polarized and begin to form a ball-like structure. EC cells are the precursors to somatic embryos that ultimately lead to whole-plant formation [8]. In most dicots, such as cotton [9], cassava [10], sweet pepper [11], and cacao [12], the ability to achieve whole-plant regeneration through SE is limited to only a few select genotypes with drastic differences in embryo formation frequencies and time to embryo formation.

Previous work has identified genes and transcription factors whose expressions are required to achieve the transition from NEC to EC cells [3,13,14]. In several monocot species, such as rice and maize, studies have shown that ectopic expression of transcription factors with an inducible promoter such as *BABY BOOM (BBM)*, *WUSCHEL* (*WUS*), and *LEAFY COTYLEDON 1* (*LEC1*) improves the embryo formation frequency in semi-recalcitrant and recalcitrant genotypes [15,16,17]. In dicots, the overexpression of *BBM* has been demonstrated to improve the embryo formation frequency in tobacco [18], sweet pepper [11], and cacao [12], but not to the same degree of efficiency as the monocot systems, suggesting that our knowledge of the genes and transcriptional pathways that are involved in SE in dicot plants remains limited.

As with most plants, SE in upland cotton (*Gossypium hirsutum* L.) is limited to only a few genotypes within the species. It has been nearly four decades since successful SE was reported. Various studies have screened genotypes for their ability to regenerate but have only found a handful of genotypes with regenerative capacity [19,20,21]. The most widely used public genotype for transformation and regeneration is Coker312 [22,23]. In another study, the genotype YZ-1 was identified as a line with higher regeneration efficiency [24]. However, both the Coker312 and YZ-1 genotypes were developed 20–30 years ago and are without suitable agronomic traits for gene function studies in cotton. More recently, a non-recalcitrant genotype, named Jin668, was described, which has a high frequency of embryo formation (~96%) with only a short duration of 45–60 days from callus initiation to the formation of ball-shaped structures [25]. Our group recently determined and characterized the global gene expression profiles of this elite regeneration line at two primary developmental stages (NEC and EC) [26]. We discovered significant transcriptome-wide differences between the two developmental stages and sharp upregulation of the key transcription factors identified in EC cells that may have a primary role in reprogramming in this genotype during SE [26].

Whole-plant regeneration through SE offers significant and biological advantages. For instance, genome editing through engineered nucleases [27] or the CRISPR-Cas9 technologies [28] offer approaches to directly modify the genome of living organisms. In plants, these powerful technologies provide a means for direct trait enhancement of elite breeding material, offering unprecedented opportunities to improve crop breeding approaches, for example, with reduced breeding cycle times, tailored trait genetics, and the potential for much larger genetic gains [29,30]. The promises of these technologies offer world-changing outcomes. However, major limitations, such as the delivery of the genome editing reagents and genotype-specific recalcitrance to regeneration, remain primary obstacles preventing widespread use in crop improvement [29,31].

In the present study, we analyzed the transcriptional profiles of NEC and EC cells harvested from the semi-recalcitrant genotype Coker312 and compared these data to the non-recalcitrant genotype Jin668 [26]. Our objectives were to determine the gene expression profile differences and similarities between Jin668 and Coker312 that fall into the following categories: (i) genes uniquely expressed in each genotype, (ii) genes with similar expression profiles, and (iii) genes with different expression profiles between the two genotypes. The results from this study will provide new opportunities to discover genes and regulatory networks involved in somatic embryogenesis that can be leveraged to develop new strategies to avert genotype-specific recalcitrance to regeneration in dicot species.

## 2. Results

### 2.1. RNAseq of Coker312 at NEC and EC Developmental Stages

Differential gene expression analysis revealed a total of 196 genes upregulated and 109 genes downregulated, respectively, in Coker312 EC when compared to Coker312 NEC, with at least 2-fold abundance difference and an adjusted *p*-value of 0.001, Supplemental Table S1.

**Figure 1:**
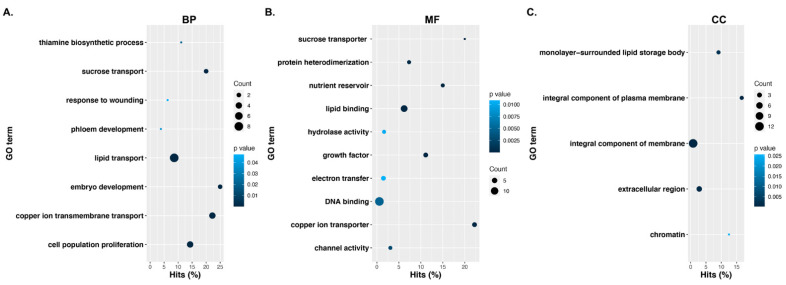
Functional enrichment of the upregulated genes in Coker312 EC cells (**A**) in the biological process (BP), (**B**) in the molecular functions, and (**C**) in the cellular components.

Genes without known orthologs or predicted functional domains in model systems, such as *Arabidopsis*, were among the most differentially expressed (~seven-fold), as shown in Table 1. Important genes with functional annotations that were largely upregulated in EC cells included lipid transfer proteins, homeobox protein 31, homeobox-3 genes, early nodulin-like proteins, copper transporters, seed gene1, and *APETALA2 (AP2)* transcription factors, as shown in Table 1 and Supplemental Table S1. Genes with the largest downregulated expression profile (almost four-fold) during the transition to EC cells included mevalonate diphosphate decarboxylase 1, conserved peptide upstream open reading frame 9, serine protease inhibitors, expansin-like proteins, and nodulin transporter family proteins, as shown in Table 1 and Supplemental Table S1. Functional enrichment of the upregulated genes in Coker312 EC cells identified the biological processes involved in the biosynthesis of vitamins, such as thiamine; the transport of sucrose, copper, and lipids; and genes involved in embryo development, as shown in Figure 1 and Supplemental Table S2. In the molecular function category, the largest number of enriched genes was categorized as transcription factors (14), followed by lipid binding (8), growth factors (4), transporters (4), and hydrolase activity (3), as shown in Supplemental Table S2.

Gene enrichment analysis of the downregulated genes in EC callus in Coker312 revealed only a handful of enriched functional categories (Supplemental Table S2). Downregulated enriched functional categories were only represented by a few genes (<4) and included the response to biotic stimulus, response to defense and wounding, hydrolase activity, and a few others (Supplemental Table S3).

### 2.2. Comparison of Coker312 to Jin668 at NEC and EC Stages

**Figure 2:**
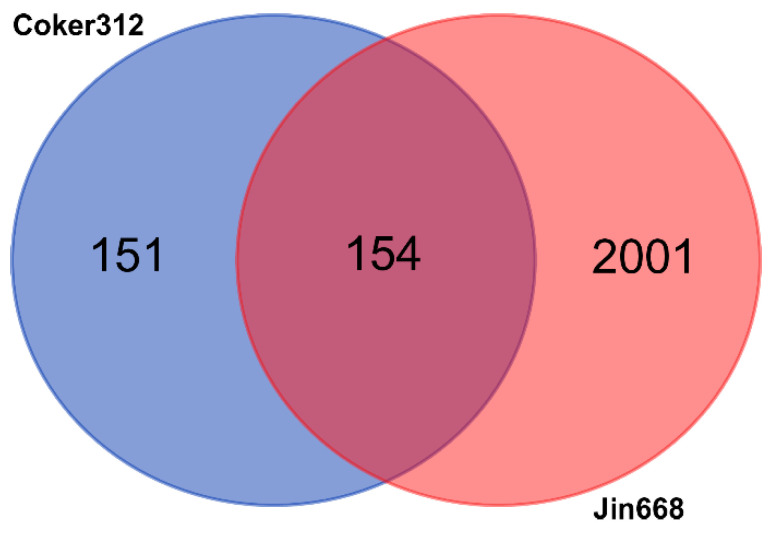
A Venn diagram representing gene expressions that are common and unique in Coker312 and Jin668 at the NEC and EC stages.

We compared the gene expression profiles of the semi-recalcitrant genotype, Coker312, to the previous transcriptomic study of a non-recalcitrant genotype, Jin668 [26]. Comparative analysis of the differentially expressed genes with at least a two-fold change profile and an error corrected *p*-value of 0.001 identified 151 unique genes in Coker312 and 2001 unique genes in Jin668 (Figure 2), with a total of 305 and 2155 differentially expressed genes in Coker312 and Jin668, respectively (Figure 2). Grouping of the 2001 genes in Jin668 revealed the Cytochrome p450 family as the most abundant group (42 members), followed by lipid transfer (35 member), helix-loop-helix DNA-binding (32 members), aquaporins (29 members), MYB transcription factors (26 members), and AP2 (Supplemental Table S4). Aside from MYBs, we also discovered additional transcription factors, such as WRKY1 (16 members), bZIPs (12 members), PLATZ (5 members), and GATAs (3 members), as shown in Supplemental Table S4. We also identified genes related to histone maintenance, MADS-box genes, and methyltransferase (Supplemental Table S4).

**Figure 3:**
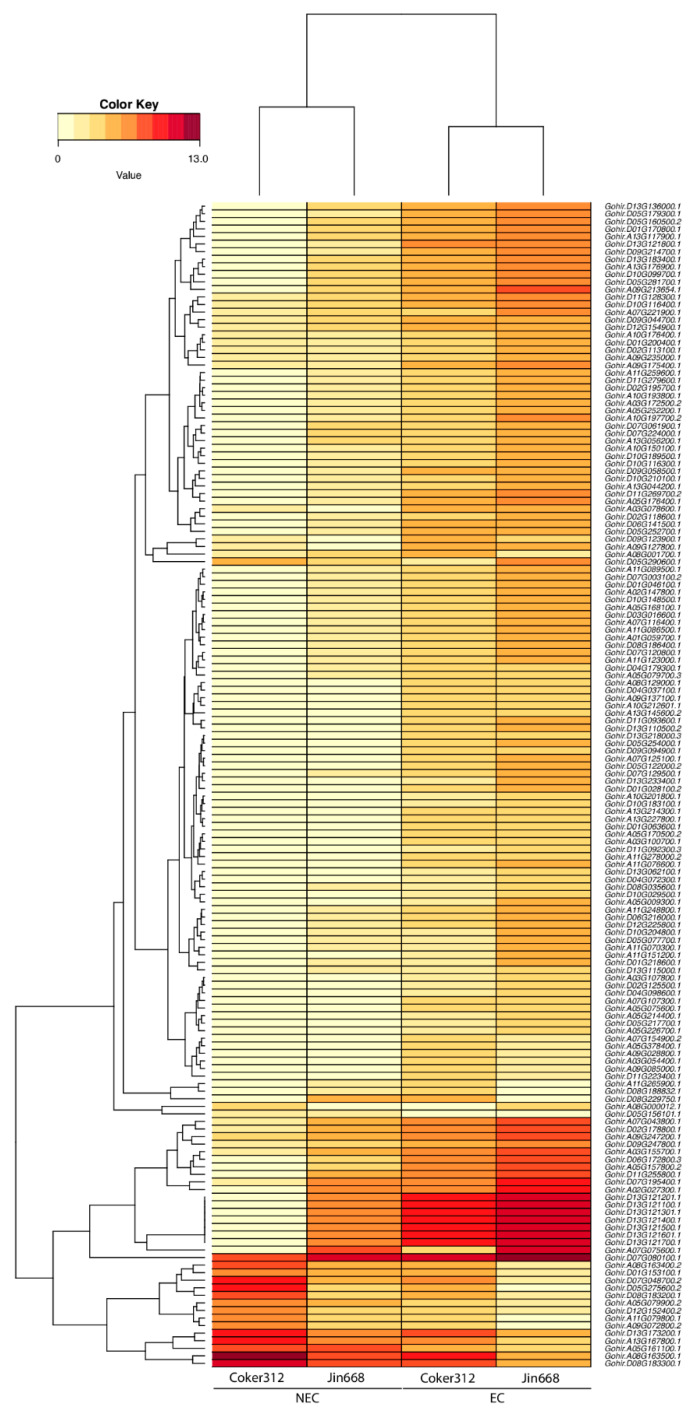
Heatmap representing the gene expression of 154 differentially expressed genes in Coker312 and Jin668 in the NEC and EC callus cells.

The 154 genes that are differentially expressed in both genotypes are of particular interest. Interestingly, a duplicate gene pair (homoeologous gene copies on both the A and D subgenomes) with the highest expression values among each genotype was comprised of a bifunctional inhibitor of lipid transfer, followed by a tandemly duplicated gene on chromosome 13 of the d-subgenome with an unknown annotation, as shown in Figure 3 and Supplemental Table S5. Other genes with high expressions in EC cells in both genotypes are lipid transfer proteins, genes with homeobox domains, and genes involved with histone proteins, as shown in Supplemental Table S5. A clustering analysis of the genes based on their expression profiles showed aggregation by condition and not genotype, as shown in Figure 3.

**Figure 4:**
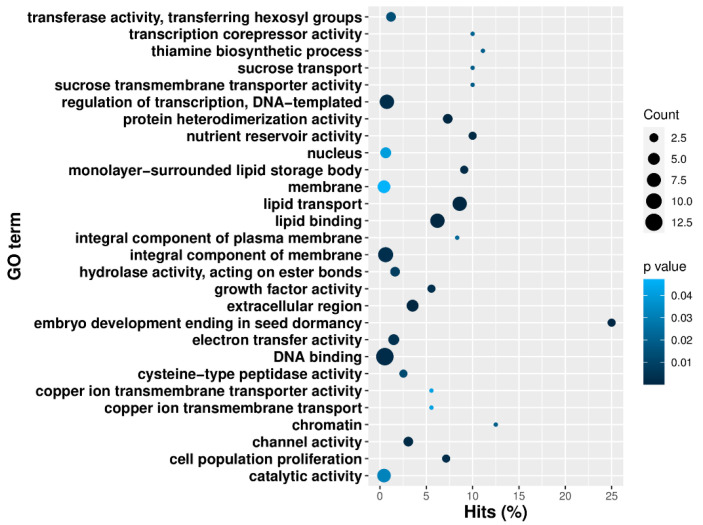
Gene ontology enrichment of the significantly upregulated genes in Coker312 in the EC cells.

Functional gene enrichment of the 154 overlapping differentially expressed genes identified genes in enriched categories, such as lipid transport, embryo development, regulation of transcription, sugar transport, vitamin biosynthesis, growth factor activity, DNA binding, cell population proliferation, and others, as shown in Supplemental Table S6 and Figure 4. We also observed that, among the 154 overlapping genes, gene expression profiles were typically higher in Jin668 EC cells versus Coker312 EC cells, as shown in Figure 3. For example, the clusters of genes in Jin668 EC had a higher expression more often than those in Coker EC, as shown in Figure 3.

**Figure 5:**
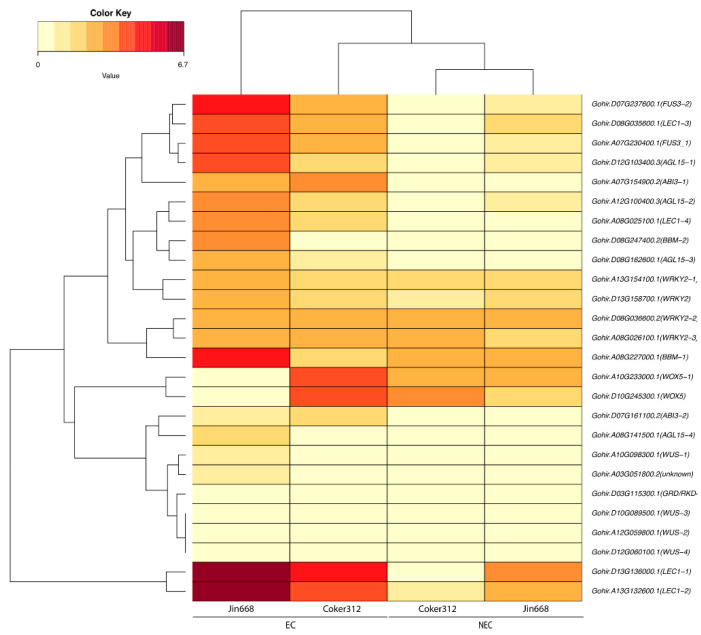
Heatmap representing the gene expression of key embryogenic genes of Coker312 and Jin668 in the NEC and EC callus cells.

We also examined the expression profiles of genes known to have a role in somatic embryogenesis, such as *BBM*, *WUS*, *LEC1*, *WUSCHEL-RELATED HOMEOBOX 5 (WOX5)*, *FUSCA3 (FUS3)*, and several other genes [26]. The genes with the sharpest fold changes were Gohir.D13G136000.1(*LEC1−1*), Gohir.A13G132600.1(*LEC1−2*), Gohir.D07G237600.1(*FUS3−2*), Gohir.D08G035600.1(*LEC1−3*), and Gohir.A07G230400.1(*FUS3_1*) in Coker312 EC and Jin668 EC, although the expression was higher in Jin668 EC in comparison of Coker312 EC, as shown in Figure 5. However, Gohir.A08G227000.1(*BBM−1*) showed a high expression in Jin668 EC, while a much lower expression was observed in Coker312 EC. Two other interesting genes, Gohir.A10G233000.1(*WOX5−1*) and Gohir.D10G245300.1(*WOX5*), were upregulated in Coker312 EC, Coker312 NEC, and Jin668 NEC, while they were almost off in Jin668 EC, as shown in Figure 5. Several important transcription factors previously reported to have a role in somatic embryogenesis, such as Gohir.D03G115300.1(*GRD*/*RKD*), Gohir.D10G089500.1(*WUS−3*), Gohir.A12G059800.1(*WUS−2*), and Gohir.D12G060100.1(*WUS−4*), showed very little to no expression in either the genotype or developmental stages, as shown in Figure 5. In the Coker312, the highest upregulated gene was *LEC1*, followed by *WOX5*, *ABSCISIC ACID INSENSITIVE (ABI3)*, *FUS3*, and *WRKY2*. In Jin668, *LEC1*, *BBM*, *FUS3*, and *AGAMOUS-LIKE15* (*AGL15*) were the highly expressed genes, and their expression was several times higher in comparison to Coker312. Surprisingly, all the copies of *WUS* were either off or very lowly expressed in either the genotype or developmental stage.

### 2.3. RT-qPCR and Validation of RNAseq

**Figure 6:**
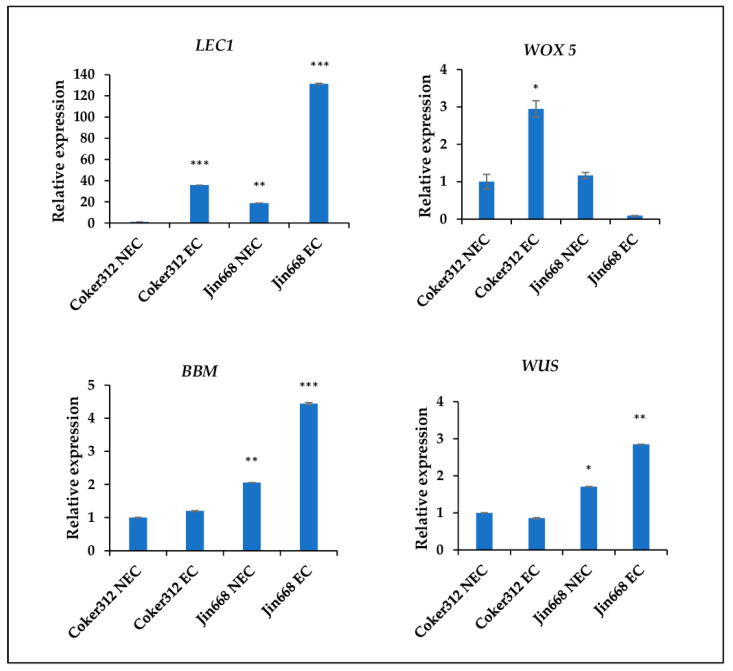
Expression of embryogenesis related gens in the non-embryogenic cells (NEC) and embryogenic cells (EC) of Coker312 and Jin668 by RT-qPCR analysis. The relative expressions of embryogenesis-related gens *GhLEC1*, *GhWOX5*, *GhBBM*, and *GhWUS* were measured. Three biological replicates and three technical replicates were used for statistical analysis. Error bars indicate ±SE (n = 3). The ΔΔCt method was used for qPCR analysis. Asterisks indicate statistically significant differences compared with the Coker312 NEC: Student’s *t* test; * *p* < 0.05; ** *p* < 0.01; *** *p* < 0.001.

RT-qPCR analysis of four critical embryogenesis genes were performed to validate the RNA-seq data, as shown in Supplemental Table S7. The relative expressions of *GhBBM*, (Gohir.D08G247400.1), *GhLEC1* (Gohir.D13G136000.1), *GhWOX5* (Gohir.D10G245300.1), and *GhWUS* (Gohir.D10G089500.1) were measured in the NEC and EC stages calli of Coker312 and Jin668, and results are presented in Figure 6. Consistent with the RNAseq data, *LEC1* was the most highly upregulated in Jin668 EC cells, followed by Coker312 EC, Jin668 NEC, and Coker312 NEC, respectively. *WOX5* gene expression was the highest in Coker312 EC cells, followed by Jin668 NEC, Coker312 NEC, and Jin668 EC, respectively. Interestingly, *BBM*, an important embryogenesis gene was downregulated in the EC and NEC cells of Coker312, while it was highly upregulated in Jin668 EC cells. In Coker312 NEC and Jin668 NEC cells, *BBM* expression was very low. RT-qPCR data also validated *WUS* expression in Coker 312 and Jin668. In comparison to the other embryogenesis genes, *WUS* expression was low in Jin668 EC cells, while it was mostly off in Jin668 NEC, Coker 312 NEC, and Coker312 EC cells. The results of RT-qPCR showed similar expression patterns of embryogenesis genes as the RNAseq data and confirmed the RNAseq results.

## 3. Discussion

Upland cotton is one of the most important economic crops worldwide and produces the largest source of renewable textile fiber. However, cotton is highly restricted to genetic improvement via transformation and whole-plant regeneration through somatic embryogenesis mainly because of the somaclonal variation in tissue culture, long in vitro regeneration via tissue culture, decline in vigor, and low potency of embryogenesis [24]. Moreover, regenerative capacity is highly genotype-dependent, and previous investigations on regenerable genotypes in cotton have not yielded many significant advancements [23,32]. In several monocot species, such as rice and maize, somatic embryogenesis has been examined at the transcriptional levels in both recalcitrant and semi-recalcitrant species. These studies have identified several key transcription factors, such as *BBM*, *WUS2*, *LEC1*, and *LEC2*, that have initiated somatic embryo formation when ectopically expressed in recalcitrant genotypes, although the frequencies and time to embryo formation still remain low and slow, respectively [12,17,33,34].

A recent study revealed an upland cotton genotype, Jin668 with elite somatic regeneration properties, such as a high frequency of embryo formation (~96%) and rapid time to cellular differentiation (45–60 days), that was developed through successive regeneration acclimation (SRA) [25]. The authors hypothesized that the regenerative potential (totipotency) is a trait that is encoded in the genome, but is epigenetically suppressed in most genotypes, leading to genotype-specific recalcitrance [25]. As a follow-up, we compared the global gene expression at two key developmental stages (NEC and EC) in the Jin668 genotype to identify the genes necessary for cellular reprogramming and the transition to EC cells [26]. We identified a sharp upregulation of several transcription factors that likely have a major role in regulating the shift from NEC to EC with subgenome bias in this allotetraploid species [26].

In this study, we collected transcriptome data from the semi-recalcitrant genotype Coker312. Coker312 is considered semi-recalcitrant because of its long time to embryo formation (90–120 days) and low frequency of embryo formation (<15%). The most upregulated genes in Coker312 EC cells are annotated as lipid transfer proteins (LTPs), homeobox-3 genes, *AP2* transcription factors, early nodulin-like proteins, and copper transporters. Previous studies have demonstrated that the LTPs are involved in the formation of a protective layer of cutin in the cell wall, surrounding the young embryo, and are implicated in the initiation of somatic embryogenesis [35]. LTPs are also abundantly expressed in the epidermis of developing tissues and play an important role in fiber elongation [36]. Earlier studies have also identified *AP2*, a super-family transcription factor, that may have a role in callus formation [37], and also contributes to biotic and abiotic stress resistance in cotton [38]. In addition, some APETALA 2/ethylene-responsive element binding factors (*AP2*/*ERFs*) are implicated in growth and developmental processes mediated by growth hormones such as gibberellins (GAs), cytokinins (CTK), and brassinosteroids (BRs) [39]. *AP2*/*ERFs* may also have a role in the hormone sensing and signaling pathways important to cellular reprogramming during the transition from NEC to EC in upland cotton, as demonstrated by the results presented here.

Our previous data identified several thousand differentially expressed genes during the transition from NEC to EC in the non-recalcitrant genotype Jin668 [26]. Comparative transcriptome analysis between Jin668 and the semi-recalcitrant genotype, Coker312, could provide a much smaller ‘candidate set’ of key genes by examining the overlap between the two genotypes. In the EC calli of both Coker312 and Jin668, the d-subgenome-encoded homolog of *LEC1* had the highest expression, indicating subgenome expression bias and a primary role in somatic embryogenesis in upland cotton. *LEC1* was described as a master regulator that shapes embryo development in *Arabidopsis* [40]. In other studies, *LEC1* has been described as a central regulator, controlling different parts of embryo morphogenesis and photosynthesis as well as seed development [41]. *WUS* is a morphogenic regulator that has been shown to induce or stimulate cellular differentiation in a range of species, such as *Arabidopsis* [16], *Zea mays* [34], and *Medicago* [42]. In cotton, the *WUS* gene had little to no expression in either genotype and developmental stage, suggesting that other genes have a more primary role in stimulating the transition from NEC to EC cells. In contrast, *WOX5* may have a more primary role than *WUS* in cotton SE. *WOX5* is a transcription factor that has been shown to be a regulator of a pool of pluripotent stem cells in the apical meristem [43] and has endowed gain-of-function mutants with somatic embryo formation in *Arabidopsis* [16]. *WOX5* is expressed during different stages of embryogenesis and post-germination growth stages [44,45]. In both Coker312 and Jin668 NEC cells, *WOX5* displays a moderate expression level and is upregulated even further in Coker312 EC cells. However, *WOX5* is downregulated in Jin668 EC cells, which suggests that it may be an upstream regulator of cellular reprogramming and that early expression of this gene is necessary for the transition to embryo formation. Earlier studies have reported that *BBM* plays an important role in transcription of *LEC1*, *LEC2*, *ABI3*, and *FUS3* [34]. In Jin668, *BBM* is highly expressed, while in Coker312, it shows less expression. Critical difference in the expression of morphogenic regulator *BBM* may be one of the reasons for the different regenerative ability of both genotypes. The genes *ABI3* and *FUS3* are also expressed in EC callus of both upland cotton genotypes (Figure 5) [26]. These genes are transcription factors of *LAFL* genes [33]. The ectopic expression of *ABI3* did not result in successful embryo development, but it has a reported role in embryo programming, being activated by *BBM* [33]. The ectopic expression of *FUS3* results in cotyledon-likes leaves, while *LEC1* and *LEC2* overexpression results in the spontaneous development of somatic embryos turning into plantlets [46,47]. In Coker312, the most highly upregulated genes in EC cells are a tandem array of genes on chromosome D13 with expression profiles that are mostly off in NEC. We also observed several genes that were highly upregulated in EC cells that have only recently been implicated with somatic embryogenesis, such as early nodulin-like protein 3 [48] and copper transporters. The upregulation of these genes has been described as a stress response in regeneration systems [49].

In Jin668, we observed a large number of unique genes (2001, Figure 2) that are also important to discuss. The most abundant group was the Cytochrome p450 family, which are important proteins that participate in the metabolism of most plant growth regulators (PGRs) [50], which may be under epigenetic control [51]. Lipid transfer was the second most abundant category, and these genes have been shown to strongly correlate with early morphogenic processes [52]. Transcription factors, including MYBs, helix-loop-helix, AP2, WRKY1, bZIPs, PLATZ, and GATAs, were also in abundance in Jin668. The expression of each of these transcription factors (except PLATZ) has been implicated in the induction of somatic embryogenesis in previous studies [53,54,55,56,57]. However, the list is quite extensive, and comparisons with Coker312 can identify a shorter list of conserved candidate genes critical to SE in cotton.

Comparative analysis of the commonly expressed genes in Coker312 and Jin668 discovered few new genes, such as growth-regulating factor 2 *(GRF2)*, Late Embryogenesis Abundant 4–5 *(LEA4-5),* and Late Embryogenesis Abundant protein *(LEA)* family protein. Previous work has shown that *GRF2* is strongly expressed in the developing tissues of the shoot apical meristem in the upper stems and root tips. Further, *GRF2* is required for the coordination of cell division and differentiation during leaf development in *Arabidopsis* [58]. *LEA* are a large group of hydrophilic proteins that play a major role in drought stress tolerance in upland cotton and are required for normal growth and development. These proteins are mostly expressed during abiotic stresses, such as in cold, drought, and high-salinity conditions [59,60], but may function in callus cells in response to the tissue-culture microenvironment.

In plant transformation and regeneration systems, both frequency and time to embryo formation are critical factors. In agrobacterium-mediated transformation, it is important to note that agrobacterium stress and selection pressure results in a reduction in embryo formation efficiency and an increase in the duration to form an embryo when compared to simply regenerating a whole plant without transformation. In Jin668 and Coker312, this stress typically adds ~8 weeks. However, embryo formation frequency in Jin668 still remains high (>80%), while, in Coker312, embryo formation drops to less than 15%, and the probability of transformed plants thorough SE is very low. In part, this may be due to differences in the expression levels of the key genes between the two genotypes. When analyzing the global trends in gene expression, a general upregulation of nearly the same transcription factors was found, but with a much higher expression profile in Jin668.

## 4. Materials and Methods

### 4.1. Plant Material and Callus Growth Conditions

The semi-recalcitrant genotype used in this study, Coker312, was obtained from the USDA Crop Germplasm Collection, College Station, TX. Seeds were surface sterilized and cultured in germination bottles on germination media [26] and kept in the dark for 7 d. The hypocotyls were excised from the 7-day-old, aseptic, etiolated seedlings, cut into 5–7 mm pieces, and cultured as explants on callus induction media containing Murashige and Skoog (MS) salts (MS basal salts mixture; PhytoTechnology Laboratories, Lenexa, KS, USA, catalog no. M524), B5 vitamins, 3.0% (*w*/*v*) glucose, 0.1 mg L^−1^ 2,4-D, 0.1 mg L^−1^ kinetin, 0.1 g L^−1^ myoinositol, 1.0 g L^−1^ MgCl2, pH 5.8, 0.26% (*w*/*v*) phytagel (PhytoTechnology Laboratories, Lenexa, KS, USA). At different developmental stages (NEC (35 d) and EC (80 d)), the callus cultures were tested for 10 days in the growth chamber. These callus cultures are part of a larger spaceflight experiment and were transferred to the Space Life Sciences Lab (SLSL: https://www.spaceflorida.gov/facilities/space-life-sciences-lab/, accessed on 25 January 2021) by automobile for growth, observation, and harvesting during the science verification testing (SVT) component of the larger experimental evaluation of somatic embryogenesis of cotton in micro-gravity. The SVT evaluates the growing conditions in a growth chamber that mimics the conditions of the Advanced Plant Habitat (APH) in orbit at the International Space Station (ISS). The conditions used for these callus cultures included: 28 ± 1 °C, 16 h (day)/8 h (night) photoperiod, 1000 ppm CO_2_, with light provided by cool-white fluorescent lamps at an irradiation of 60 μmol m^−2^s^−1^ and 50% relative humidity. Plates were rotated on alternate days for equal light distribution. After testing for 10 days in the growth chamber, whole calli were harvested at two different stages, i.e., NEC (45-day-old calli) and EC (90-day-old calli with ball-shaped embryo structures) into RNA*later* stabilization and storage solution. Whole calli were kept at room temperature for 24 h and moved to −80 °C for longer storage.

### 4.2. RNAseq

A total of four biological replications for each stage were used for mRNA sequencing. Total RNA was extracted from Coker312 callus material following the guanidine thiocyanate method described by the authors of [61]. RNA integrity and concentration were assayed on an BioAnalyzer2100 (Agilent) and considered high quality with RNA Integrity Number (RIN) values ≥ 7 and total masses (≥2.0 µg total RNA) for all biological replicates. Sequencing libraries were prepared following the standard protocols of the Illumina TruSeq Stranded RNA kit. Transcriptome sequences were collected on an Illumina NovaSeq to a depth of at least 40 million read pairs per replicate sample. Raw sequences were preprocessed to remove adapter and low-quality bases with Trimmomatic software v.0.38 [61]. Cleaned reads were mapped to the *Gossypium hirsutum* (TM1 v.2.0) reference assembly [62] using the Bowtie2 short-read aligner [63]. Transcript abundance was quantified with RSEM [64], and differentially expressed transcripts were determined with edgeR [65]. Because cotton is an allotetraploid species with highly identical subgenomes, genes are expected to be in multiple copies and may be expressed with bias at the subgenome level [62]. It is important to note that genes described in Figure 5 were assigned a gene name, and the annotated gene and its expression value were derived from primary transcripts of the V2.0 assembly described by Chen et al., 2020 [62].

### 4.3. Reverse Transcription-Quantitative PCR (RT-qPCR)

Reverse transcription-quantitative PCR (RT-qPCR) was carried out to validate the RNAseq data [66]. Four differentially expressed embryogenesis genes (*GhLEC1*, *GhWOX5*, *GhBBM*, and *GhWUS*) and three biological reps of each NEC and EC stage callus of Coker312 and JIn668 were used for the RT-qPCR. In total, 1 μg of the total RNAs was used to synthesize the first strand of cDNA using the M-MuLV reverse transcriptase (New England Biolabs, USA) and primed by d(T)25-VN as per the manufacturer’s instructions. RT-qPCR of gene transcripts was carried out on an iCycler iQ system (Bio-Rad, Hercules, CA, USA) in 20 μL of PCR reaction solution using the Luna Universal qPCR Master Mix, New England Biolabs, USA. Thermal cycling conditions comprised of initial denaturation at 95 °C for 60 s, followed by 40 cycles of 95 °C for 20 s, 62 °C for 20 s, and 72 °C for 20 s. Finally, a unique melting curve was performed from 55.0 °C to 95.0 °C in 0.5 °C increments to amplify a unique PCR product. Two reference genes, *GhPP2A1* [67] and *GhUBQ7* [25], were used to normalize the expression data. The Ct values of three technical samples for each of the three biological replicates were used to calculate the relative expression of genes using the 2^−ΔΔCt^ equation [68]. All primer pairs, except for the reference genes, were designed from the conserved coding sequence and listed in Supplemental Table S7.

## 5. Conclusions

In this study, comparative transcriptome profiling of two upland cotton genotypes that differ in regenerative capacity and developmental timing revealed a short list of candidate genes whose expression and expression abundance are critical for somatic embryogenesis in upland cotton. The genes *LEC1*, *BBM*, *FUS3*, *AGL15*, *ABI3*, and *WOX5* were commonly expressed in Coker312 and Jin668 EC cells. These results provide a foundation for candidate gene testing (in various combinations) for their role in initiating somatic embryogenesis in recalcitrant cotton genotypes.

## Figures and Tables

**Table 1:** Top 20 up-/down-regulated genes in Coker312 EC cells.

Genes Upregulated in EC Cells					
Gene	log2(EC)	log2(NEC)	logFC	Gene Function	Best Hit *Arabidopsis*
Gohir.D13G121100.1	7.250014115	0.19660704	7.05		NA
Gohir.D13G121201.1	7.250014115	0.19660704	7.05		NA
Gohir.D13G121301.1	7.250014115	0.19660704	7.05		NA
Gohir.D13G121400.1	7.250014115	0.19660704	7.05		NA
Gohir.D13G121500.1	7.250014115	0.19660704	7.05		NA
Gohir.D13G121601.1	7.250014115	0.19660704	7.05		NA
Gohir.D13G121700.1	7.250014115	0.19660704	7.05		NA
Gohir.A02G027300.1	6.048672137	0.98477161	5.06	lipid transfer protein 1	AT2G38540
Gohir.D13G121800.1	4.873813198	0	4.87	NA	NA
Gohir.D11G255800.1	5.140655972	0.64431778	4.5	homeobox protein 31	NA
Gohir.D09G214700.1	4.210077099	0	4.21	lipid transfer protein 6	AT3G08770
Gohir.A13G117900.1	4.170726276	0	4.17		NA
Gohir.A05G157800.2	5.21680405	1.14990967	4.07	homeobox-3	AT2G33880
Gohir.A05G258900.2	3.996750279	0	4		NA
Gohir.D05G252700.1	4.069014678	0.14795788	3.92	sucrose-proton symporter 2	AT1G71880
Gohir.D02G178800.1	5.818876119	1.96458346	3.85	early nodulin-like protein 3	AT4G32490
Gohir.D01G170800.1	4.741520918	0.91838623	3.82	D-amino acid aminotransferase-like PLP-dependent enzymes superfamily protein	AT1G50110
Gohir.D03G120300.1	6.743972672	2.93243919	3.81	Ctr copper transporter family	AT5G59030
Gohir.D06G172800.3	4.864136609	1.0765591	3.79		NA
Gohir.D05G160500.2	4.30560579	0.5685186	3.74	homeobox-3	AT2G33880
**Genes Downregulated in EC Cells**					
**Gene**	**log2(EC)**	**log2(NEC)**	**logFC**	**Gene Function**	**Best Hit *Arabidopsis***
Gohir.A08G000012.1	0	2.61917822	−2.62	NA	NA
Gohir.A08G035700.1	0	2.65351867	−2.65	conserved peptide upstream open reading frame 9	AT3G25572
Gohir.D11G135400.1	0.532067552	3.19440229	−2.66	nodulin MtN21/EamA-like transporter family protein	NA
Gohir.A08G163500.1	9.153073165	11.8621445	−2.71	expansin-like B1	AT4G17030
Gohir.A08G164800.1	2.183645305	4.89442976	−2.71	expansin-like B1	AT4G17030
Gohir.A11G128400.1	0	2.72421369	−2.72	Serine protease inhibitor, potato inhibitor I-type family protein	AT2G38870
Gohir.A08G221266.1	0	2.82048534	−2.82	NA	NA
Gohir.D08G183300.1	8.199755764	11.1391459	−2.94	expansin-like B1	AT4G17030
Gohir.A06G029900.1	1.270229907	4.25149188	−2.98	Phosphoglycerate mutase family protein	AT5G64460
Gohir.A10G027250.1	1.523561956	4.53362564	−3.01		NA
Gohir.D03G000201.1	0	3.07347751	−3.07		NA
Gohir.D04G021500.1	1.379066399	4.5088723	−3.13	NA	NA
Gohir.A08G053950.1	0	3.21396933	−3.21	Cellulose synthase family protein	AT4G32410
Gohir.A05G393450.1	1.019346089	4.39882918	−3.38	NA	NA
Gohir.A09G136000.1	0.298658316	3.73487217	−3.44		NA
Gohir.D12G006350.1	0	3.47235779	−3.47	NA	NA
Gohir.A05G393425.1	0.211635253	3.73595535	−3.52	NA	NA
Gohir.D05G275600.2	4.984908294	8.57295407	−3.59	NA	NA
Gohir.D08G110800.1	0	3.62690633	−3.63	small acidic protein 1	AT4G13520
Gohir.D04G021600.1	0.633198686	4.3772626	−3.74	NA	NA

## Data Availability

All sequence data reported in this manuscript can be found in the NCBI sequence read archive (SRA) under BioProject study PRJNA747913 (Samples: SRR15186777-ARR15186784).

## Supplementary Materials

The following are available online at https://www.mdpi.com/article/10.3390/plants10091775/s1, Table S1: Differentially expressed genes in Coker312 in NEC compared to EC cells with at least 2-fold change and error corrected *p*-values less than or equal to 0.001, Table S2: Functional enrichment categories of genes upregulated in EC callus cells in Coker312, Table S3: Functional enrichment categories of genes downregulated in EC callus cells in Coker312, Table S4: Grouping of the 2001 unique genes in Jin668 based on PFAM functional domains. Table S5: Gene expression matrix (Log TMM+1) of the 154 overlapping differentially expressed genes in Coker312 and Jin668, Table S6: Functional enrichment categories of the 154 genes that are commonly differentially expressed among Coker312 and Jin668, Table S7: List of primers used for RT-qPCR of embryogenesis genes.
