# Supplementary material for: Comparative Transcriptomics of Non-Embryogenic and Embryogenic Callus in Semi-Recalcitrant and Non-Recalcitrant Upland Cotton Lines
Source: Plants (Basel). 2021 Aug 26;10(9):1775. doi: 10.3390/plants10091775 (PMC8472754; doi:10.3390/plants10091775)
Supplement: Supplementary file 1 [file plants-10-01775-s001.zip › plants-1326400-Supplementary.pdf]

plants

MDPI

Article

Comparative Transcriptomics of Non-Embryogenic and Embryogenic Callus in Semi-Recalcitrant and Non-Recalcitrant Upland Cotton Lines

Supplementary Materials

Table 1. Differentially expressed genes in Coker312 in NEC compared to EC cells with at least 2-fold change and error corrected *p*-values less than or equal to 0.001.

Gene	sample_A	sample_B	log2(exprA)	log2(exprB)	logFC	FDR	Gene Function	Best Hit Arabidopsis
Gohir.D13G121100.1	coker312_ec	coker312_nec	7.25001412	0.19660704	7.05	0.00828203		NA
Gohir.D13G121201.1	coker312_ec	coker312_nec	7.25001412	0.19660704	7.05	0.00828203		NA
Gohir.D13G121301.1	coker312_ec	coker312_nec	7.25001412	0.19660704	7.05	0.00828203		NA
Gohir.D13G121400.1	coker312_ec	coker312_nec	7.25001412	0.19660704	7.05	0.00828203		NA
Gohir.D13G121500.1	coker312_ec	coker312_nec	7.25001412	0.19660704	7.05	0.00828203		NA
Gohir.D13G121601.1	coker312_ec	coker312_nec	7.25001412	0.19660704	7.05	0.00828203		NA
Gohir.D13G121700.1	coker312_ec	coker312_nec	7.25001412	0.19660704	7.05	0.00828203		NA
Gohir.A02G027300.1	coker312_ec	coker312_nec	6.04867214	0.98477161	5.06	0.00753762	lipid transfer protein 1	AT2G38540
Gohir.D13G121800.1	coker312_ec	coker312_nec	4.8738132	0	4.87	0.00037422	NA	NA
Gohir.D11G255800.1	coker312_ec	coker312_nec	5.14065597	0.64431778	4.5	0.00923906	homeobox protein 31	NA
Gohir.D09G214700.1	coker312_ec	coker312_nec	4.2100771	0	4.21	0.00052201	lipid transfer protein 6	AT3G08770
Gohir.A13G117900.1	coker312_ec	coker312_nec	4.17072628	0	4.17	0.0004828		NA
Gohir.A05G157800.2	coker312_ec	coker312_nec	5.21680405	1.14990967	4.07	0.00010509	homeobox-3	AT2G33880
Gohir.A05G258900.2	coker312_ec	coker312_nec	3.99675028	0	4	0.0008677		NA
Gohir.D05G252700.1	coker312_ec	coker312_nec	4.06901468	0.14795788	3.92	0.00177464	sucrose-proton symporter 2	AT1G71880
Gohir.D02G178800.1	coker312_ec	coker312_nec	5.81887612	1.96458346	3.85	0.00099779	early nodulin-like protein 3	AT4G32490
Gohir.D01G170800.1	coker312_ec	coker312_nec	4.74152092	0.91838623	3.82	0.00162989	D-aminoacid aminotransferase-like PLP-dependent enzymes superfamily protein	AT1G50110
Gohir.D03G120300.1	coker312_ec	coker312_nec	6.74397267	2.93243919	3.81	0.00698967	Ctr copper transporter family	AT5G59030
Gohir.D06G172800.3	coker312_ec	coker312_nec	4.86413661	1.0765591	3.79	0.04921492		NA

Gohir.D05G160500.2	coker312_ec	coker312_nec	4.30560579	0.5685186	3.74	0.00584911	homeobox-3	AT2G33880
Gohir.D11G269700.2	coker312_ec	coker312_nec	3.95874989	0.29631056	3.66	0.00512508	glutamate dehydrogenase 1	AT5G18170
Gohir.D13G136000.1	coker312_ec	coker312_nec	4.09448945	0.45417589	3.64	0.03394927	Histone superfamily protein	AT1G21970
Gohir.A07G043800.1	coker312_ec	coker312_nec	5.88988721	2.28214323	3.61	0.03260585	oleosin 1	AT4G25140
Gohir.A07G206000.1	coker312_ec	coker312_nec	3.60169652	0	3.6	0.02303869		NA
Gohir.D05G296000.1	coker312_ec	coker312_nec	3.51500592	0	3.52	0.00034346	Serine protease inhibitor, potato inhibitor I-type family protein	NA
Gohir.D11G128300.1	coker312_ec	coker312_nec	4.92186473	1.41196851	3.51	0.00867374	Cupredoxin superfamily protein	AT5G26330
Gohir.D05G179300.1	coker312_ec	coker312_nec	4.13035434	0.64616266	3.48	0.00823764	nuclear factor Y, subunit B6	AT5G47670
Gohir.A05G265200.2	coker312_ec	coker312_nec	3.47443604	0	3.47	0.03161829	sequence-specific DNA binding transcription factors;transcription regulators	AT5G50011
Gohir.D02G073600.1	coker312_ec	coker312_nec	3.43602855	0	3.44	0.00063732		NA
Gohir.D09G058500.1	coker312_ec	coker312_nec	3.98577364	0.54596837	3.44	0.04410495	seed gene 1	AT4G26740
Gohir.D07G195400.1	coker312_ec	coker312_nec	5.54170068	2.10936056	3.43	0.03781717	Glycine-rich protein family	NA
Gohir.A03G172500.2	coker312_ec	coker312_nec	3.71072379	0.3021728	3.41	1.98E-05	Bifunctional inhibitor/lipid-transfer protein/seed storage 2S albumin superfamily protein	AT5G64080
Gohir.D11G093600.1	coker312_ec	coker312_nec	3.66868647	0.26423615	3.4	0.00338363	plasma membrane intrinsic protein 1;4	AT1G01620
Gohir.D10G210100.1	coker312_ec	coker312_nec	3.72650429	0.34141652	3.39	0.00555047	Papain family cysteine protease	AT3G54940
Gohir.D04G037100.1	coker312_ec	coker312_nec	3.40653667	0.02431968	3.38	0.00014496	protodermal factor 1	NA
Gohir.D05G281700.1	coker312_ec	coker312_nec	4.06935846	0.69331968	3.38	0.0374621	Peroxidase superfamily protein	AT5G05340
Gohir.A03G143950.1	coker312_ec	coker312_nec	3.3704433	0	3.37	1.80E-15	NA	NA
Gohir.D11G114750.1	coker312_ec	coker312_nec	3.37336981	0	3.37	0.03403979	NA	NA
Gohir.A10G150100.1	coker312_ec	coker312_nec	3.35162833	0	3.35	5.56E-16	lipid transfer protein 1	AT2G38540
Gohir.A05G176400.1	coker312_ec	coker312_nec	3.76139166	0.44042072	3.32	0.01530182	nuclear factor Y, subunit B6	AT5G47670
Gohir.A05G252200.1	coker312_ec	coker312_nec	3.38929146	0.11236652	3.28	0.01212026	GDSL-like Lipase/Acylhydrolase superfamily protein	AT1G71691
Gohir.D08G188832.1	coker312_ec	coker312_nec	3.25414048	0	3.25	0.00087391		NA
Gohir.A09G137100.1	coker312_ec	coker312_nec	3.21116798	0	3.21	0.0010631		AT5G06270
Gohir.D06G141500.1	coker312_ec	coker312_nec	4.04753839	0.83349734	3.21	0.00317903	chlorophyll A/B binding protein 1	AT1G29930
Gohir.A11G265900.1	coker312_ec	coker312_nec	3.644087	0.44466707	3.2	4.53E-12	nuclear factor Y, subunit B5	AT2G47810
Gohir.A03G046900.1	coker312_ec	coker312_nec	6.15394717	2.9582859	3.2	0.00027291	Ctr copper transporter family	AT5G59030
Gohir.A03G155700.1	coker312_ec	coker312_nec	4.7486759	1.59024271	3.16	0.02059974	early nodulin-like protein 3	AT4G32490
Gohir.A09G175400.1	coker312_ec	coker312_nec	4.62029315	1.47092726	3.15	0.03754469	PEBP (phosphatidylethanolamine-binding protein) family protein	AT1G18100
Gohir.D13G110500.2	coker312_ec	coker312_nec	3.3110671	0.17120683	3.14	0.03860756	Glycoprotein membrane precursor GPI-anchored	AT1G54860
Gohir.A08G129000.1	coker312_ec	coker312_nec	3.30553283	0.2003788	3.11	0.00033093	vacuolar iron transporter 1	NA
Gohir.A09G213654.1	coker312_ec	coker312_nec	4.3244506	1.21288057	3.11	0.02678008	lipid transfer protein 6	AT3G08770
Gohir.A11G259600.1	coker312_ec	coker312_nec	3.68605224	0.59263643	3.09	2.72E-05	glutamate dehydrogenase 1	AT5G18170

Gohir.A07G075600.1	coker312_ec	coker312_nec	3.09288397	0	3.09	0.00076901	Bifunctional inhibitor/lipid-transfer protein/seed storage 2S albumin superfamily protein	NA
Gohir.D09G123900.1	coker312_ec	coker312_nec	5.37107091	2.29777836	3.07	0.0158506	Late Embryogenesis Abundant 4-5	NA
Gohir.A13G176900.1	coker312_ec	coker312_nec	4.0498921	0.99131776	3.06	0.03289862	nodulin MtN21 /EamA-like transporter family protein	AT1G09380
Gohir.D13G183400.1	coker312_ec	coker312_nec	4.18237449	1.14469903	3.04	0.00326601	nodulin MtN21 /EamA-like transporter family protein	AT1G09380
Gohir.A10G212601.1	coker312_ec	coker312_nec	3.0259147	0	3.03	0.00090496	Protein of unknown function (DUF674)	NA
Gohir.A10G197700.2	coker312_ec	coker312_nec	3.04054179	0.02998287	3.01	0.00154981	cytochrome P45, family 77, subfamily A, polypeptide 4	AT5G04660
Gohir.D11G279600.1	coker312_ec	coker312_nec	3.2298955	0.26183166	2.97	0.0281318	alpha/beta-Hydrolases superfamily protein	AT3G05600
Gohir.D09G053232.1	coker312_ec	coker312_nec	2.95958471	0	2.96	0.00115512	Cyclin/Brf1-like TBP-binding protein	AT3G09360
Gohir.D02G195700.1	coker312_ec	coker312_nec	3.37559554	0.41575867	2.96	0.00748124	Bifunctional inhibitor/lipid-transfer protein/seed storage 2S albumin superfamily protein	AT5G64080
Gohir.D08G229750.1	coker312_ec	coker312_nec	2.96162333	0	2.96	0.02665124	Ribosomal protein L18ae/LX family protein	AT2G34480
Gohir.D10G189500.1	coker312_ec	coker312_nec	2.94091845	0	2.94	0.00091451	annexin 8	AT5G12380
Gohir.A04G100800.1	coker312_ec	coker312_nec	3.42586209	0.48233202	2.94	0.0015703	CCR-like	AT3G26740
Gohir.A05G378400.1	coker312_ec	coker312_nec	2.93187213	0	2.93	0.00088372	protodermal factor 1	NA
Gohir.D09G064700.1	coker312_ec	coker312_nec	2.9292229	0	2.93	0.04031876	small acidic protein 1	AT4G13520
Gohir.D04G179300.1	coker312_ec	coker312_nec	3.31542132	0.39944456	2.92	0.0266772	tryptophan aminotransferase of Arabidopsis 1	AT1G70560
Gohir.A10G193800.1	coker312_ec	coker312_nec	3.28614187	0.37962096	2.91	0.00055864	Integrase-type DNA-binding superfamily protein	AT3G54320
Gohir.A07G154900.2	coker312_ec	coker312_nec	3.0731347	0.169925	2.9	0.01607601	AP2/B3-like transcriptional factor family protein	AT3G24650
Gohir.A13G044200.1	coker312_ec	coker312_nec	3.97416217	1.07792658	2.9	8.48E-06	basic helix-loop-helix (bHLH) DNA-binding superfamily protein	AT3G19500
Gohir.A05G167366.1	coker312_ec	coker312_nec	2.87656606	0	2.88	0.00051886	photosystem II subunit Q-2	NA
Gohir.D11G223400.1	coker312_ec	coker312_nec	2.87519029	0	2.88	0.00400539	heat shock factor binding protein	AT4G15802
Gohir.D09G166000.1	coker312_ec	coker312_nec	9.91895713	7.04898812	2.87	1.52E-07	Gibberellin-regulated family protein	AT4G09600
Gohir.A09G028800.1	coker312_ec	coker312_nec	3.02715405	0.16221004	2.86	0.0071134	Oleosin family protein	AT3G01570
Gohir.A13G145600.2	coker312_ec	coker312_nec	3.88176249	1.0306892	2.85	3.21E-10	AGAMOUS-like 8	NA
Gohir.D01G028100.2	coker312_ec	coker312_nec	2.851799	0	2.85	0.00099779	Uncharacterised protein family (UPF497)	AT1G17200
Gohir.D07G061900.1	coker312_ec	coker312_nec	3.04666567	0.21412481	2.83	0.03180584	strictosidine synthase 3	NA
Gohir.D07G210200.1	coker312_ec	coker312_nec	2.76595901	0	2.77	5.04E-11	translocase of outer membrane 22-V	AT5G43970
Gohir.A13G176050.1	coker312_ec	coker312_nec	2.76807844	0	2.77	0.00074222		NA
Gohir.D06G216000.1	coker312_ec	coker312_nec	2.75702325	0	2.76	0.00121422		AT1G12064
Gohir.A07G218200.1	coker312_ec	coker312_nec	9.08320273	6.32614182	2.76	0.00254165	Gibberellin-regulated family protein	NA
Gohir.A11G123000.1	coker312_ec	coker312_nec	3.27545409	0.51702367	2.76	0.03899655	Cupredoxin superfamily protein	AT5G26330
Gohir.A08G194050.1	coker312_ec	coker312_nec	2.75017771	0	2.75	0.00111005	Preprotein translocase Sec, Sec61-beta subunit protein	AT5G60460

Gohir.D05G260150.1	coker312_ec	coker312_nec	2.7456676	0	2.75	0.00340841	Arabidopsis defensin-like protein	NA
Gohir.D07G120800.1	coker312_ec	coker312_nec	3.17903326	0.4254593	2.75	0.04786331	RmlC-like cupins superfamily protein	AT2G28490
Gohir.A07G128000.1	coker312_ec	coker312_nec	2.90996507	0.17632277	2.73	0.00127046		NA
Gohir.D07G224000.1	coker312_ec	coker312_nec	2.87911759	0.16607268	2.71	0.01273688	ABC transporter family protein	AT3G28345
Gohir.D02G118600.1	coker312_ec	coker312_nec	3.69465737	0.98622888	2.71	0.04571241	beta-tonoplast intrinsic protein	AT1G17810
Gohir.D07G129500.1	coker312_ec	coker312_nec	2.86868677	0.17887396	2.69	1.24E-06	Integrase-type DNA-binding superfamily protein	AT1G51190
Gohir.D09G044700.1	coker312_ec	coker312_nec	4.97947664	2.29219277	2.69	0.01071094	subtilase 1.3	AT5G51750
Gohir.A11G248800.1	coker312_ec	coker312_nec	2.86532499	0.18142064	2.68	0.00190315	ATPase E1-E2 type family protein / haloacid dehalogenase-like hydrolase family protein	AT3G22910
Gohir.A05G075600.1	coker312_ec	coker312_nec	2.66902677	0	2.67	0.00108441		NA
Gohir.D11G258032.1	coker312_ec	coker312_nec	2.66789213	0	2.67	0.00187003	Cyclin/Brf1-like TBP-binding protein	NA
Gohir.D10G116300.1	coker312_ec	coker312_nec	2.62784027	0	2.63	4.95E-06	lipid transfer protein 3	AT5G59320
Gohir.D10G116400.1	coker312_ec	coker312_nec	4.37079201	1.75403232	2.62	0.00019784	lipid transfer protein 1	AT2G38540
Gohir.A07G125100.1	coker312_ec	coker312_nec	2.69019358	0.08270259	2.61	3.47E-05	Integrase-type DNA-binding superfamily protein	AT1G51190
Gohir.D03G016600.1	coker312_ec	coker312_nec	3.13750352	0.5290713	2.61	0.00524752	UDP-glucosyl transferase 72E1	AT3G50740
Gohir.A09G127800.1	coker312_ec	coker312_nec	4.91456452	2.30392684	2.61	0.02264651	Late Embryogenesis Abundant 4-5	NA
Gohir.A10G074000.1	coker312_ec	coker312_nec	2.60264691	0	2.6	0.00121422		NA
Gohir.D05G254000.1	coker312_ec	coker312_nec	2.72770267	0.12432814	2.6	0.03405204	GDSL-like Lipase/Acylhydrolase superfamily protein	AT1G71691
Gohir.A01G162700.2	coker312_ec	coker312_nec	3.72027847	1.12697286	2.59	2.88E-07	photosystem I light harvesting complex gene 2	AT3G61470
Gohir.A13G214300.1	coker312_ec	coker312_nec	2.62550429	0.03421572	2.59	0.00032815	fatty acid desaturase 2	AT3G12120
Gohir.A07G116400.1	coker312_ec	coker312_nec	3.12697286	0.56071495	2.57	0.03329521	RmlC-like cupins superfamily protein	AT2G28490
Gohir.D13G218000.3	coker312_ec	coker312_nec	2.76574689	0.1928254	2.57	0.034783	fatty acid desaturase 2	AT3G12120
Gohir.A11G076600.1	coker312_ec	coker312_nec	3.0583165	0.5290713	2.53	3.40E-05	homeobox-leucine zipper protein 3	AT3G60390
Gohir.D01G046100.1	coker312_ec	coker312_nec	3.274858	0.76298656	2.51	0.00031435	UDP-Glycosyltransferase superfamily protein	AT5G54010
Gohir.A13G056200.1	coker312_ec	coker312_nec	3.17919254	0.68347189	2.5	0.000361	alpha/beta-Hydrolases superfamily protein	NA
Gohir.A13G227800.1	coker312_ec	coker312_nec	2.49620667	0	2.5	0.00133398	WUSCHEL related homeobox 2	AT5G59340
Gohir.D10G099700.1	coker312_ec	coker312_nec	3.67964899	1.18205661	2.5	0.02421225	AINTEGUMENTA-like 6	AT5G10510
Gohir.D01G063600.1	coker312_ec	coker312_nec	2.48954294	0	2.49	0.001384	germin-like protein 5	NA
Gohir.D05G122000.2	coker312_ec	coker312_nec	2.52079937	0.03421572	2.49	0.00273232	NA	NA
Gohir.A03G078600.1	coker312_ec	coker312_nec	3.809826	1.34766566	2.46	0.04855444	beta-tonoplast intrinsic protein	AT1G17810
Gohir.A07G107300.1	coker312_ec	coker312_nec	2.4486367	0	2.45	0.00129599	PHYTOSULFOKINE 3 PRECURSOR	AT3G44735
Gohir.D04G098600.1	coker312_ec	coker312_nec	2.45180456	0	2.45	0.00140214		AT3G52610
Gohir.A11G086500.1	coker312_ec	coker312_nec	3.20742437	0.75702325	2.45	0.01480963		NA
Gohir.D06G092766.1	coker312_ec	coker312_nec	3.45890691	1.0335111	2.43	0.00154232		NA
Gohir.D08G186400.1	coker312_ec	coker312_nec	3.17504549	0.75017771	2.42	0.03368901	Homeobox-leucine zipper protein 4 (HB-4) / HD-ZIP protein	AT4G16780
Gohir.A11G151200.1	coker312_ec	coker312_nec	2.41386483	0	2.41	5.56E-16	NA	NA

Gohir.D03G112500.1	coker312_ec	coker312_nec	2.50080205	0.09356018	2.41	0.00050967	hydroxysteroid dehydrogenase 1	AT5G50600
Gohir.A03G107800.1	coker312_ec	coker312_nec	2.41386483	0	2.41	0.00157387	phytosulfokine 4 precursor	NA
Gohir.A09G085000.1	coker312_ec	coker312_nec	2.93941466	0.54201036	2.4	0.00124093	Late embryogenesis abundant protein (LEA) family protein	NA
Gohir.A09G149900.1	coker312_ec	coker312_nec	3.2298955	0.82781902	2.4	0.00260607		NA
Gohir.D11G210700.1	coker312_ec	coker312_nec	2.93772103	0.53306492	2.4	0.02303869	phytosulfokine 4 precursor	NA
Gohir.A08G234500.1	coker312_ec	coker312_nec	2.85219864	0.45943162	2.39	0.0078605	Protein of unknown function (DUF64)	AT2G42610
Gohir.A13G111375.1	coker312_ec	coker312_nec	2.78764141	0.40925515	2.38	0.0012208	GATA transcription factor 15	AT3G06740
Gohir.A11G186000.1	coker312_ec	coker312_nec	3.37323058	0.99783433	2.38	0.01180178	RmlC-like cupins superfamily protein	AT2G18540
Gohir.D13G233400.1	coker312_ec	coker312_nec	2.40163047	0.02998287	2.37	0.00356751	WUSCHEL related homeobox 2	AT5G59340
Gohir.D09G057166.1	coker312_ec	coker312_nec	2.368489	0	2.37	0.04698718	ubiquitin-specific protease 21	AT4G17895
Gohir.D09G094900.1	coker312_ec	coker312_nec	2.63737839	0.27500705	2.36	0.00377464	Basic-leucine zipper (bZIP) transcription factor family protein	NA
Gohir.A10G125800.1	coker312_ec	coker312_nec	5.00773372	2.64477922	2.36	0.01156253		NA
Gohir.D12G225800.1	coker312_ec	coker312_nec	2.69532574	0.33227828	2.36	0.0230335	growth-regulating factor 2	AT2G22840
Gohir.D13G062100.1	coker312_ec	coker312_nec	2.45733163	0.12961274	2.33	9.07E-05	glutamine synthase clone R1	AT5G37600
Gohir.D02G125500.1	coker312_ec	coker312_nec	2.32883646	0	2.33	0.00198235	NAC (No Apical Meristem) domain transcriptional regulator superfamily protein	AT1G26870
Gohir.D03G120400.1	coker312_ec	coker312_nec	2.57628026	0.25701062	2.32	0.03225584	copper transporter 1	AT5G59030
Gohir.D10G204800.1	coker312_ec	coker312_nec	2.45127706	0.12961274	2.32	0.04579118	cytochrome P45, family 77, subfamily A, polypeptide 4	AT5G04660
Gohir.A09G247200.1	coker312_ec	coker312_nec	5.51733869	3.20851726	2.31	0.00214968	Heavy metal transport/detoxification superfamily protein	NA
Gohir.A10G201800.1	coker312_ec	coker312_nec	2.45417589	0.15833703	2.3	0.00705051	Papain family cysteine protease	AT3G54940
Gohir.A11G282630.1	coker312_ec	coker312_nec	2.27917411	0	2.28	0.00273998	NA	NA
Gohir.D06G078100.1	coker312_ec	coker312_nec	3.39272956	1.12498977	2.27	0.01014328	Integrase-type DNA-binding superfamily protein	AT1G19210
Gohir.D05G077700.1	coker312_ec	coker312_nec	2.35867757	0.0976108	2.26	0.00513322	tetraspanin3	AT5G60220
Gohir.D10G029500.1	coker312_ec	coker312_nec	2.40844014	0.17120683	2.24	5.75E-05	homeobox-3	AT2G33880
Gohir.A06G003600.1	coker312_ec	coker312_nec	2.79347976	0.5518851	2.24	0.00253593	tubulin beta-1 chain	AT1G75780
Gohir.D10G183100.1	coker312_ec	coker312_nec	2.37823416	0.15055968	2.23	0.01071094	pyruvate dehydrogenase kinase	AT3G06483
Gohir.D13G115000.1	coker312_ec	coker312_nec	2.34255475	0.1256511	2.22	0.0003778	calreticulin 1a	AT1G56340
Gohir.A05G251100.1	coker312_ec	coker312_nec	2.25580284	0.03421572	2.22	0.0040055	sucrose-proton symporter 2	AT1G22710
Gohir.A03G100700.1	coker312_ec	coker312_nec	2.69220399	0.46780116	2.22	0.03961243	NAC (No Apical Meristem) domain transcriptional regulator superfamily protein	AT1G26870
Gohir.D05G068200.1	coker312_ec	coker312_nec	4.84136935	2.63250089	2.21	6.87E-05	selenium binding	NA
Gohir.A11G089500.1	coker312_ec	coker312_nec	2.86473092	0.65718266	2.21	0.01882697	Major facilitator superfamily protein	AT4G00370
Gohir.A01G059700.1	coker312_ec	coker312_nec	3.29366476	1.09288397	2.2	0.0003778	UDP-Glycosyltransferase superfamily protein	AT4G27560
Gohir.D01G113800.1	coker312_ec	coker312_nec	3.09169983	0.90612095	2.19	7.37E-05	HXXXD-type acyl-transferase family protein	AT3G23840

Gohir.D11G297300.2	coker312_ec	coker312_nec	3.79888001	1.62386686	2.18	0.00060985	Histone H3 K4-specific methyltransferase SET7/9 family protein	NA
Gohir.A03G054400.1	coker312_ec	coker312_nec	2.82944368	0.64984535	2.18	0.0339767	hydroxysteroid dehydrogenase 1	AT5G50600
Gohir.A05G079700.3	coker312_ec	coker312_nec	3.21288057	1.03210084	2.18	0.03754469	Pollen Ole e 1 allergen and extensin family protein	AT2G27385
Gohir.A05G214400.1	coker312_ec	coker312_nec	2.16542962	0	2.17	0.00216238	amino acid permease 6	AT5G49630
Gohir.A07G187550.1	coker312_ec	coker312_nec	2.17024556	0	2.17	0.00268323	NA	NA
Gohir.D08G252650.1	coker312_ec	coker312_nec	2.17024556	0	2.17	0.00268323	NA	NA
Gohir.D12G161400.1	coker312_ec	coker312_nec	2.16928366	0	2.17	0.00476711	phytosulfokine 4 precursor	NA
Gohir.D07G080100.1	coker312_ec	coker312_nec	9.51970504	7.34775423	2.17	0.03953611	Bifunctional inhibitor/lipid-transfer protein/seed storage 2S albumin superfamily protein	NA
Gohir.D01G200400.1	coker312_ec	coker312_nec	3.83794324	1.67536431	2.16	0.00225725	GDSL-like Lipase/Acylhydrolase family protein	AT5G03820
Gohir.D07G003100.2	coker312_ec	coker312_nec	2.70043972	0.54497988	2.16	0.02756583	pleiotropic drug resistance 1	AT3G30842
Gohir.A05G226700.1	coker312_ec	coker312_nec	2.15023471	0	2.15	0.00224184		AT1G78170
Gohir.D02G113100.1	coker312_ec	coker312_nec	3.68380872	1.54497988	2.14	0.00805021	PHYTOCYSTATIN 2	NA
Gohir.D10G152600.2	coker312_ec	coker312_nec	2.26002566	0.14274017	2.12	2.25E-07	AUX/IAA transcriptional regulator family protein	AT5G43700
Gohir.A12G111400.1	coker312_ec	coker312_nec	3.8623522	1.74717254	2.12	9.46E-07	thylakoid rhodanese-like	AT4G01050
Gohir.A02G147800.1	coker312_ec	coker312_nec	2.81782788	0.69599381	2.12	0.0020329	HAESA-like 2	NA
Gohir.A08G033700.1	coker312_ec	coker312_nec	7.72520264	5.61067054	2.11	3.73E-09		AT5G41761
Gohir.A05G408700.1	coker312_ec	coker312_nec	4.13840493	2.03174806	2.11	6.46E-06	plant intracellular ras group-related LRR 6	AT2G19330
Gohir.A03G004200.1	coker312_ec	coker312_nec	6.0674236	3.96023367	2.11	8.49E-05		AT3G01670
Gohir.A03G142800.1	coker312_ec	coker312_nec	10.9666821	8.8772872	2.09	0.0095051		NA
Gohir.A09G235000.1	coker312_ec	coker312_nec	4.16583156	2.09017595	2.08	0.00058097	Haem oxygenase-like, multi-helical	AT3G16990
Gohir.D03G116400.2	coker312_ec	coker312_nec	5.90710698	3.83116788	2.08	0.00416514	YELLOW STRIPE like 3	AT5G53550
Gohir.A05G170500.2	coker312_ec	coker312_nec	2.66471038	0.58688481	2.08	0.03225584	Protein of unknown function, DUF584	AT4G21970
Gohir.A07G221900.1	coker312_ec	coker312_nec	3.68929916	1.61117238	2.08	0.03910744	myb domain protein 118	NA
Gohir.A11G115700.1	coker312_ec	coker312_nec	2.17919254	0.09895848	2.08	0.04096591	Glutathione S-transferase family protein	AT3G62760
Gohir.A11G207600.1	coker312_ec	coker312_nec	2.07382023	0	2.07	0.03808151		NA
Gohir.A06G023200.1	coker312_ec	coker312_nec	2.06004738	0	2.06	0.00451494	Glycosyl hydrolase family 35 protein	NA
Gohir.D12G154900.1	coker312_ec	coker312_nec	4.38328957	2.3262497	2.06	0.01831834	high mobility group A	NA
Gohir.A05G168100.1	coker312_ec	coker312_nec	2.61447428	0.55385197	2.06	0.04735392	thiazole biosynthetic enzyme, chloroplast (ARA6) (THI1) (THI4)	AT5G54770
Gohir.D05G217700.1	coker312_ec	coker312_nec	2.23327454	0.18396283	2.05	0.00018257	amino acid permease 6	AT5G49630
Gohir.D09G247800.1	coker312_ec	coker312_nec	4.81480674	2.76913698	2.05	0.01204628	Heavy metal transport/detoxification superfamily protein	AT5G63530
Gohir.A11G278000.2	coker312_ec	coker312_nec	2.79618241	0.74588269	2.05	0.02596155	F-box family protein	NA
Gohir.D02G166100.1	coker312_ec	coker312_nec	10.9038261	8.86142815	2.04	0.00099779		NA

Gohir.D01G218600.1	coker312_ec	coker312_nec	2.03597574	0	2.04	0.0030288	Basic-leucine zipper (bZIP) transcription factor family protein	NA
Gohir.A05G009300.1	coker312_ec	coker312_nec	2.03667915	0	2.04	0.0030288	Ran BP2/NZF zinc finger-like superfamily protein	AT3G15680
Gohir.A11G070300.1	coker312_ec	coker312_nec	2.04159345	0	2.04	0.00350625		NA
Gohir.D13G065501.1	coker312_ec	coker312_nec	2.28540222	0.24488706	2.04	0.00404358	GTP binding	AT1G30580
Gohir.D11G056400.1	coker312_ec	coker312_nec	2.73573878	0.71369581	2.02	0.00027997	DNAJ-like 2	NA
Gohir.D11G248600.1	coker312_ec	coker312_nec	4.73074863	2.7132559	2.02	0.00075447		NA
Gohir.A10G176400.1	coker312_ec	coker312_nec	3.81803247	1.80074462	2.02	0.00667984	pyruvate dehydrogenase kinase	AT3G06483
Gohir.D11G092300.3	coker312_ec	coker312_nec	2.64431778	0.6210555	2.02	0.01084745	AT hook motif DNA-binding family protein	AT3G61310
Gohir.D04G072300.1	coker312_ec	coker312_nec	2.23143241	0.21536797	2.02	0.01764647	Fatty acid hydroxylase superfamily	AT1G02205
Gohir.D05G359200.1	coker312_ec	coker312_nec	2.01042181	0	2.01	9.06E-05	protochlorophyllide oxidoreductase A	AT5G54190
Gohir.D08G164100.2	coker312_ec	coker312_nec	2.01399815	0	2.01	0.00463541		NA
Gohir.A08G001700.1	coker312_ec	coker312_nec	4.90245953	2.90323113	2	6.28E-05	copper transporter 1	AT2G26975
Gohir.D11G250000.3	coker312_ec	coker312_nec	2.11470017	0.11236652	2	0.0028811	hydroxyproline-rich glycoprotein family protein	NA
Gohir.D10G148500.1	coker312_ec	coker312_nec	2.64938553	0.65260122	2	0.00347497	Major facilitator superfamily protein	AT2G32040
Gohir.D07G161100.2	coker312_ec	coker312_nec	1.47664091	0.04684025	1.43	0.02450128	AP2/B3-like transcriptional factor family protein	AT3G24650
Gohir.D02G179700.2	coker312_ec	coker312_nec	1.52105074	3.52230689	-2	2.19E-05	FAD/NAD(P)-binding oxidoreductase family protein	AT3G04650
Gohir.D12G008000.2	coker312_ec	coker312_nec	4.48626386	6.48907725	-2	0.00521328		NA
Gohir.A12G155732.1	coker312_ec	coker312_nec	0.90380956	2.90592848	-2	0.00822479	NA	NA
Gohir.D01G142300.1	coker312_ec	coker312_nec	3.6794238	5.69382146	-2.01	1.30E-06	RNA polymerase I subunit 43	AT1G60620
Gohir.A09G186001.1	coker312_ec	coker312_nec	0	2.00611846	-2.01	0.0070295		NA
Gohir.A06G132900.1	coker312_ec	coker312_nec	2.80714881	4.81608766	-2.01	0.01147665		AT1G29270
Gohir.A04G084700.1	coker312_ec	coker312_nec	7.98503927	10.003747	-2.02	5.27E-05		NA
Gohir.A07G058400.1	coker312_ec	coker312_nec	2.38128337	4.40388121	-2.02	0.00223119	BSD domain-containing protein	AT1G69030
Gohir.A05G150400.1	coker312_ec	coker312_nec	4.67242534	6.69789889	-2.03	1.69E-15	purine permease 1	AT4G18210
Gohir.D05G028300.1	coker312_ec	coker312_nec	5.32088176	7.36043451	-2.04	5.62E-07	O-Glycosyl hydrolases family 17 protein	AT5G56590
Gohir.D03G008800.1	coker312_ec	coker312_nec	2.62620548	4.66544962	-2.04	0.0001614	Major facilitator superfamily protein	AT2G16660
Gohir.D01G153100.1	coker312_ec	coker312_nec	5.29487802	7.34672647	-2.05	1.48E-07	NA	NA
Gohir.D05G290600.1	coker312_ec	coker312_nec	1.96014098	4.01167453	-2.05	0.03392159	Nucleotide-diphospho-sugar transferases superfamily protein	AT5G03760
Gohir.D04G141000.1	coker312_ec	coker312_nec	4.21870368	6.27526784	-2.06	1.77E-05	FKBP-like peptidyl-prolyl cis-trans isomerase family protein	AT5G13410
Gohir.A06G160700.1	coker312_ec	coker312_nec	2.57240465	4.62859864	-2.06	0.00050249		AT3G05390
Gohir.D06G127800.1	coker312_ec	coker312_nec	3.7131459	5.7692957	-2.06	0.00715333		AT4G21445
Gohir.A04G116200.1	coker312_ec	coker312_nec	1.73248629	3.78899885	-2.06	0.01031403	Class I glutamine amidotransferase-like superfamily protein	AT2G23970
Gohir.D07G048700.2	coker312_ec	coker312_nec	6.61603416	8.67386378	-2.06	0.03374924	NA	NA

Gohir.D08G192500.2	coker312_ec	coker312_nec	4.63674061	6.71069625	-2.07	1.92E-06	Protein of unknown function (DUF1442)	AT2G45360
Gohir.D05G217000.1	coker312_ec	coker312_nec	3.04281941	5.11765349	-2.07	0.01006382		AT4G37810
Gohir.D10G038001.1	coker312_ec	coker312_nec	3.55520263	5.62342795	-2.07	0.01188177	Curculin-like (mannose-binding) lectin family protein	AT1G78860
Gohir.D07G077600.1	coker312_ec	coker312_nec	0	2.06557231	-2.07	0.04274229		NA
Gohir.A11G241700.1	coker312_ec	coker312_nec	0.66993384	2.74846123	-2.08	8.39E-05		NA
Gohir.D12G152400.2	coker312_ec	coker312_nec	3.79815423	5.90093924	-2.1	3.23E-08	Galactose oxidase/kelch repeat superfamily protein	AT1G67480
Gohir.A03G188800.1	coker312_ec	coker312_nec	2.4246534	4.52023365	-2.1	0.00089044	Protein of unknown function (DUF1685)	AT4G33985
Gohir.A12G104000.1	coker312_ec	coker312_nec	5.0653138	7.17359719	-2.11	0.00049089	phloem protein 2-A13	AT3G61060
Gohir.D05G268133.1	coker312_ec	coker312_nec	0	2.11636476	-2.12	2.25E-07	NA	NA
Gohir.D06G128800.1	coker312_ec	coker312_nec	7.98800294	10.1035921	-2.12	0.00157387	glycine-rich protein	AT4G21620
Gohir.D05G147400.1	coker312_ec	coker312_nec	1.92827556	4.0536327	-2.13	0.00049367	NA	NA
Gohir.A07G093500.1	coker312_ec	coker312_nec	6.69318027	8.82795237	-2.13	0.01784725		NA
Gohir.A03G213900.1	coker312_ec	coker312_nec	4.87921564	7.00510801	-2.13	0.0459223		NA
Gohir.A08G119300.1	coker312_ec	coker312_nec	5.18070484	7.32273037	-2.14	0.00088372	Adenine nucleotide alpha hydrolases-like superfamily protein	AT3G62550
Gohir.A13G205400.1	coker312_ec	coker312_nec	4.28347734	6.42760617	-2.14	0.00098093	2-oxoglutarate (2OG) and Fe(II)-dependent oxygenase superfamily protein	NA
Gohir.D10G176700.1	coker312_ec	coker312_nec	3.82690435	5.96730697	-2.14	0.00180015		NA
Gohir.D09G053166.1	coker312_ec	coker312_nec	0	2.14404637	-2.14	0.00305586	transportin 1	AT2G16950
Gohir.D11G246301.1	coker312_ec	coker312_nec	2.4903134	4.63110428	-2.14	0.01953011	NA	NA
Gohir.D12G270900.1	coker312_ec	coker312_nec	3.71281585	5.86059041	-2.15	0.00166425		NA
Gohir.A03G214000.1	coker312_ec	coker312_nec	5.39930784	7.55289192	-2.15	0.00652299		NA
Gohir.A03G058800.1	coker312_ec	coker312_nec	0	2.15088457	-2.15	0.02990558		AT4G25225
Gohir.D05G219300.1	coker312_ec	coker312_nec	3.93073734	6.08890482	-2.16	0.00056893	Phosphoglycerate mutase family protein	AT5G64460
Gohir.A02G171300.1	coker312_ec	coker312_nec	3.68403323	5.84358192	-2.16	0.00232468	Major facilitator superfamily protein	AT2G16660
Gohir.D10G165400.1	coker312_ec	coker312_nec	5.85486838	8.0230557	-2.17	8.80E-07	Peroxidase superfamily protein	AT2G39040
Gohir.A01G001900.2	coker312_ec	coker312_nec	3.05779682	5.22677824	-2.17	4.37E-05	ACT-like protein tyrosine kinase family protein	AT4G38470
Gohir.A08G224300.1	coker312_ec	coker312_nec	0	2.16542962	-2.17	0.00141277		AT1G10865
Gohir.D06G033600.1	coker312_ec	coker312_nec	1.93922658	4.1215128	-2.18	3.00E-05	MATE efflux family protein	AT5G65380
Gohir.D11G245000.1	coker312_ec	coker312_nec	2.06591692	4.24328825	-2.18	0.00866201	myb domain protein 5	AT3G13540
Gohir.D10G014400.2	coker312_ec	coker312_nec	2.12664253	4.31208426	-2.19	0.00124542	beta glucosidase 46	AT1G61820
Gohir.A07G010900.1	coker312_ec	coker312_nec	3.57349572	5.77396996	-2.2	0.00115169	beta-galactosidase 12	AT3G13750
Gohir.A01G057200.1	coker312_ec	coker312_nec	7.7538385	9.95119145	-2.2	0.0014987		NA
Gohir.D07G013000.1	coker312_ec	coker312_nec	1.7895206	3.98832113	-2.2	0.04313168	homolog of CFIM-25	AT4G29820
Gohir.A13G167800.1	coker312_ec	coker312_nec	6.97766917	9.19243338	-2.21	0.00025283	threonine aldolase 1	AT1G08630
Gohir.A06G123900.1	coker312_ec	coker312_nec	7.69073834	9.90121386	-2.21	0.00139669	glycine-rich protein	AT4G21620

Gohir.D01G096800.1	coker312_ec	coker312_nec	1.85877675	4.08720823	-2.23	2.83E-06	Protein kinase superfamily protein	AT4G35030
Gohir.A12G270500.1	coker312_ec	coker312_nec	3.96689185	6.21225804	-2.25	0.0001073		NA
Gohir.A05G289200.1	coker312_ec	coker312_nec	0.76128527	3.00880956	-2.25	0.02760048	Nucleotide-diphospho-sugar transferases superfamily protein	AT5G03760
Gohir.D13G050500.1	coker312_ec	coker312_nec	0	2.26062791	-2.26	0.03961243		NA
Gohir.D12G166200.1	coker312_ec	coker312_nec	1.16800012	3.45048546	-2.28	3.95E-05		NA
Gohir.A05G161100.1	coker312_ec	coker312_nec	5.36075054	7.66312399	-2.3	0.00099779	arabinogalactan protein 3	NA
Gohir.D05G156101.1	coker312_ec	coker312_nec	0	2.31266517	-2.31	0.00157298		NA
Gohir.D10G248000.1	coker312_ec	coker312_nec	0.29865832	2.61659295	-2.32	0.03191845	Protein of unknown function (DUF1313)	AT2G29950
Gohir.D13G121033.1	coker312_ec	coker312_nec	0	2.32969769	-2.33	0.03284103		NA
Gohir.D13G173200.1	coker312_ec	coker312_nec	7.49738882	9.83449159	-2.34	0.00021484	threonine aldolase 1	AT1G08630
Gohir.D11G112600.1	coker312_ec	coker312_nec	2.58976349	4.93950869	-2.35	0.00333291	myb domain protein 2	AT3G06490
Gohir.A01G231300.1	coker312_ec	coker312_nec	3.48065289	5.84471204	-2.36	0.01187405	Cystatin/monellin superfamily protein	AT5G47550
Gohir.A02G056100.1	coker312_ec	coker312_nec	1.63273353	4.00495076	-2.37	0.01630992	myb domain protein 78	AT3G06490
Gohir.D01G043600.1	coker312_ec	coker312_nec	7.39789706	9.79033546	-2.39	0.00017349		NA
Gohir.A09G072800.2	coker312_ec	coker312_nec	3.99376492	6.39639928	-2.4	4.77E-10	Protein of unknown function (DUF56)	AT4G32480
Gohir.A05G079900.2	coker312_ec	coker312_nec	3.80756101	6.20504056	-2.4	0.00039307		NA
Gohir.D05G229650.1	coker312_ec	coker312_nec	0.19408705	2.59000312	-2.4	0.01480585		NA
Gohir.D07G004000.1	coker312_ec	coker312_nec	0	2.41359408	-2.41	0.03915303	ROTUNDIFOLIA like 12	NA
Gohir.A05G319400.1	coker312_ec	coker312_nec	4.07596041	6.50331693	-2.43	0.00111745		NA
Gohir.D01G142100.1	coker312_ec	coker312_nec	1.87970577	4.31846147	-2.44	3.80E-06	PLANT CADMIUM RESISTANCE 2	AT1G14870
Gohir.A11G079800.1	coker312_ec	coker312_nec	4.06160341	6.49671799	-2.44	0.00112166	Plant invertase/pectin methylesterase inhibitor superfamily	AT2G45220
Gohir.D08G108700.1	coker312_ec	coker312_nec	0.8253786	3.26333494	-2.44	0.00622449	chitinase A	AT5G24090
Gohir.D06G029000.1	coker312_ec	coker312_nec	2.20976527	4.65895407	-2.45	1.37E-06	Phosphoglycerate mutase family protein	AT5G64460
Gohir.A02G052400.1	coker312_ec	coker312_nec	4.97471285	7.43800169	-2.46	6.77E-07	MLP-like protein 423	NA
Gohir.D08G183200.1	coker312_ec	coker312_nec	5.23890148	7.70327377	-2.46	0.00108941	expansin-like B1	AT4G17030
Gohir.D01G097650.1	coker312_ec	coker312_nec	0	2.47300757	-2.47	8.16E-06	NA	NA
Gohir.D05G082800.2	coker312_ec	coker312_nec	3.34738219	5.82792062	-2.48	0.00166425		NA
Gohir.D11G107733.1	coker312_ec	coker312_nec	0	2.4918531	-2.49	0.00378632	NA	NA
Gohir.A08G163400.2	coker312_ec	coker312_nec	5.53032049	8.09068938	-2.56	0.00034008	expansin-like B1	AT4G17030
Gohir.A10G037200.2	coker312_ec	coker312_nec	5.21109009	7.77011545	-2.56	0.00123182	Curculin-like (mannose-binding) lectin family protein	AT1G78830
Gohir.D05G028500.1	coker312_ec	coker312_nec	1.93130484	4.49230186	-2.56	0.0055054	profilin 5	AT2G19770
Gohir.A05G021200.1	coker312_ec	coker312_nec	0	2.57191946	-2.57	0.00063241	ROTUNDIFOLIA like 12	AT4G13395
Gohir.D05G022600.1	coker312_ec	coker312_nec	0	2.57191946	-2.57	0.00063241	ROTUNDIFOLIA like 12	AT4G13395
Gohir.A11G234000.1	coker312_ec	coker312_nec	1.64293257	4.23450132	-2.59	0.00027693	myb domain protein 5	AT3G13540
Gohir.D02G235500.1	coker312_ec	coker312_nec	4.90674632	7.50081798	-2.59	0.03575131		NA

Gohir.D10G063000.1	coker312_ec	coker312_nec	0	2.60573137	-2.61	3.46E-07	mevalonate diphosphate decarboxylase 1	NA
Gohir.A08G000012.1	coker312_ec	coker312_nec	0	2.61917822	-2.62	0.00070966	NA	NA
Gohir.A08G035700.1	coker312_ec	coker312_nec	0	2.65351867	-2.65	0.04137655	conserved peptide upstream open reading frame 9	AT3G25572
Gohir.D11G135400.1	coker312_ec	coker312_nec	0.53206755	3.19440229	-2.66	1.69E-05	nodulin MtN21 /EamA-like transporter family protein	NA
Gohir.A08G163500.1	coker312_ec	coker312_nec	9.15307316	11.8621445	-2.71	0.00011925	expansin-like B1	AT4G17030
Gohir.A08G164800.1	coker312_ec	coker312_nec	2.18364531	4.89442976	-2.71	0.00218429	expansin-like B1	AT4G17030
Gohir.A11G128400.1	coker312_ec	coker312_nec	0	2.72421369	-2.72	0.00055673	Serine protease inhibitor, potato inhibitor I-type family protein	AT2G38870
Gohir.A08G221266.1	coker312_ec	coker312_nec	0	2.82048534	-2.82	1.47E-12	NA	NA
Gohir.D08G183300.1	coker312_ec	coker312_nec	8.19975576	11.1391459	-2.94	9.50E-05	expansin-like B1	AT4G17030
Gohir.A06G029900.1	coker312_ec	coker312_nec	1.27022991	4.25149188	-2.98	3.94E-07	Phosphoglycerate mutase family protein	AT5G64460
Gohir.A10G027250.1	coker312_ec	coker312_nec	1.52356196	4.53362564	-3.01	0.01420752		NA
Gohir.D03G000201.1	coker312_ec	coker312_nec	0	3.07347751	-3.07	0.00109429		NA
Gohir.D04G021500.1	coker312_ec	coker312_nec	1.3790664	4.5088723	-3.13	0.00045562	NA	NA
Gohir.A08G053950.1	coker312_ec	coker312_nec	0	3.21396933	-3.21	1.81E-24	Cellulose synthase family protein	AT4G32410
Gohir.A05G393450.1	coker312_ec	coker312_nec	1.01934609	4.39882918	-3.38	0.01027071	NA	NA
Gohir.A09G136000.1	coker312_ec	coker312_nec	0.29865832	3.73487217	-3.44	0.00337629		NA
Gohir.D12G006350.1	coker312_ec	coker312_nec	0	3.47235779	-3.47	0.03724549	NA	NA
Gohir.A05G393425.1	coker312_ec	coker312_nec	0.21163525	3.73595535	-3.52	0.00033661	NA	NA
Gohir.D05G275600.2	coker312_ec	coker312_nec	4.98490829	8.57295407	-3.59	0.00014148	NA	NA
Gohir.D08G110800.1	coker312_ec	coker312_nec	0	3.62690633	-3.63	0.00025845	small acidic protein 1	AT4G13520
Gohir.D04G021600.1	coker312_ec	coker312_nec	0.63319869	4.3772626	-3.74	9.42E-06	NA	NA

Table 2. Functional enrichment categories of genes upregulated in EC callus cells in Coker312.

category	over_represented_pvalue	numDEInCat	numInCat	term	ontology	over_represented_FDR	go_term	gene_ids
GO:0006869	1.49E-09	8	93	lipid transport	BP	2.37E-06	BP lipid transport	Gohir.A02G027300.1, Gohir.D02G195700.1, Gohir.A10G150100.1, Gohir.D10G116300.1, Gohir.D10G116400.1, Gohir.D09G214700.1, Gohir.A07G075600.1, Gohir.D07G080100.1
GO:0035434	3.91E-07	4	18	copper ion transmembrane transport	BP	0.00016407	BP copper ion transmembrane transport	Gohir.A03G046900.1, Gohir.A08G001700.1, Gohir.D03G120300.1, Gohir.D03G120400.1
GO:0008283	3.60E-06	4	28	cell population proliferation	BP	0.00121043	BP cell population proliferation	Gohir.D12G161400.1, Gohir.A03G107800.1, Gohir.A07G107300.1, Gohir.D11G210700.1
GO:0015770	0.0001656	2	10	sucrose transport	BP	0.03089414	BP sucrose transport	Gohir.D05G252700.1, Gohir.A05G251100.1
GO:0009793	0.00032748	2	8	embryo development ending in seed dormancy	BP	0.04229486	BP embryo development ending in seed dormancy	Gohir.D09G123900.1, Gohir.A09G127800.1
GO:0009228	0.02173023	1	9	thiamine biosynthetic process	BP	1	BP thiamine biosynthetic process	Gohir.A05G168100.1
GO:0010088	0.03950169	1	26	phloem development	BP	1	BP phloem development	Gohir.A03G004200.1
GO:0009611	0.04722556	1	16	response to wounding	BP	1	BP response to wounding	Gohir.D05G296000.1
GO:0016021	0.00018974	12	1474	integral component of membrane	CC	0.03185765	CC integral component of membrane	Gohir.A03G046900.1, Gohir.A08G001700.1, Gohir.D03G120300.1, Gohir.D03G120400.1, Gohir.D07G224000.1, Gohir.A07G043800.1, Gohir.A09G028800.1, Gohir.D13G183400.1, Gohir.A13G176900.1, Gohir.D05G077700.1, Gohir.D10G148500.1, Gohir.A11G089500.1
GO:0005887	0.00028503	2	12	integral component of plasma membrane	CC	0.03988058	CC integral component of plasma membrane	Gohir.D05G252700.1, Gohir.A05G251100.1
GO:0005576	0.00101927	4	142	extracellular region	CC	0.12223918	CC extracellular region	Gohir.D12G161400.1, Gohir.A03G107800.1, Gohir.A07G107300.1, Gohir.D11G210700.1
GO:0012511	0.00251635	2	22	monolayer-surrounded lipid storage body	CC	0.2816638	CC monolayer-surrounded lipid storage body	Gohir.A07G043800.1, Gohir.A09G028800.1
GO:0000785	0.02567395	1	8	chromatin	CC	1	CC chromatin	Gohir.D12G154900.1

GO:0008289	2.82E-09	8	129	lipid binding	MF	2.37E-06	MF lipid binding	Gohir.A02G027300.1, Gohir.D02G195700.1, Gohir.A10G150100.1, Gohir.D10G116300.1, Gohir.D10G116400.1, Gohir.D09G214700.1, Gohir.A07G075600.1, Gohir.D07G080100.1
GO:0005375	3.91E-07	4	18	copper ion transmembrane transporter activity	MF	0.00016407	MF copper ion transmembrane transporter activity	Gohir.A03G046900.1, Gohir.A08G001700.1, Gohir.D03G120300.1, Gohir.D03G120400.1
GO:0045735	9.65E-06	3	20	nutrient reservoir activity	MF	0.00236172	MF nutrient reservoir activity	Gohir.A11G186000.1, Gohir.A07G116400.1, Gohir.D07G120800.1
GO:0008083	9.85E-06	4	36	growth factor activity	MF	0.00236172	MF growth factor activity	Gohir.D12G161400.1, Gohir.A03G107800.1, Gohir.A07G107300.1, Gohir.D11G210700.1
GO:0008515	0.0001656	2	10	sucrose transmembrane transporter activity	MF	0.03089414	MF sucrose transmembrane transporter activity	Gohir.D05G252700.1, Gohir.A05G251100.1
GO:0046982	0.00026718	3	41	protein heterodimerization activity	MF	0.03988058	MF protein heterodimerization activity	Gohir.A11G265900.1, Gohir.D05G179300.1, Gohir.A05G176400.1
GO:0015267	0.00275497	3	98	channel activity	MF	0.28909992	MF channel activity	Gohir.D02G118600.1, Gohir.A03G078600.1, Gohir.D11G093600.1
GO:0003677	0.00478533	14	2462	DNA binding	MF	0.47262182	MF DNA binding	Gohir.D12G154900.1, Gohir.A11G076600.1, Gohir.A10G193800.1, Gohir.D06G078100.1, Gohir.D10G029500.1, Gohir.D10G099700.1, Gohir.D08G186400.1, Gohir.A07G221900.1, Gohir.A07G125100.1, Gohir.D07G129500.1, Gohir.D13G233400.1, Gohir.A13G227800.1, Gohir.D02G125500.1, Gohir.A03G100700.1
GO:0016788	0.01026901	3	184	hydrolase activity, acting on ester bonds	MF	0.95786998	MF hydrolase activity, acting on ester bonds	Gohir.D05G254000.1, Gohir.A05G252200.1, Gohir.D01G200400.1
GO:0009055	0.010873	4	269	electron transfer activity	MF	0.95838331	MF electron transfer activity	Gohir.D11G128300.1, Gohir.A11G123000.1, Gohir.D02G178800.1, Gohir.A03G155700.1
GO:0016630	0.01141612	1	5	protochlorophyllide reductase activity	MF	0.95838331	MF protochlorophyllide reductase activity	Gohir.D05G359200.1
GO:0008234	0.01531986	2	79	cysteine-type peptidase activity	MF	1	MF cysteine-type peptidase activity	Gohir.A10G201800.1, Gohir.D10G210100.1

GO:0016758	0.01635015	3	252	transferase activity, transferring hexosyl groups	MF	1	MF transferase activity, transferring hexosyl groups	Gohir.D01G046100.1, Gohir.A01G059700.1, Gohir.D03G016600.1
GO:0003714	0.03077942	1	10	transcription corepressor activity	MF	1	MF transcription corepressor activity	Gohir.D11G223400.1
GO:0004867	0.0372011	1	10	serine-type endopeptidase inhibitor activity	MF	1	MF serine-type endopeptidase inhibitor activity	Gohir.D05G296000.1

Table 3. Functional enrichment categories of genes downregulated in EC callus cells in Coker312,.

category	over_represen ted_pvalue	numDEIn Cat	numInCat	term	ontology	over_represen ted_FDR	go_term	gene_ids
GO:0009607	0.00976084	2	74	response to biotic stimulus	BP	1	BP response to biotic stimulus	Gohir.A02G052400.1, Gohir.D10G176700.1
GO:0006378	0.01214819	1	8	mRNA polyadenylation	BP	1	BP mRNA polyadenylation	Gohir.D07G013000.1
GO:0006952	0.02251825	2	129	defense response	BP	1	BP defense response	Gohir.A02G052400.1, Gohir.D10G176700.1
GO:0009611	0.02830462	1	16	response to wounding	BP	1	BP response to wounding	Gohir.A11G128400.1
GO:0042753	0.0386005	1	18	positive regulation of circadian rhythm	BP	1	BP positive regulation of circadian rhythm	Gohir.D10G248000.1
GO:0005975	0.04173281	3	712	carbohydrate metabolic process	BP	1	BP carbohydrate metabolic process	Gohir.D05G028300.1, Gohir.D08G108700.1, Gohir.A07G010900.1
GO:0005576	0.0001243	4	142	extracellular region	CC	0.20870174	CC extracellular region	Gohir.D08G183300.1, Gohir.D08G183200.1, Gohir.A08G163500.1, Gohir.A08G164800.1

GO:0005849	0.01274743	1	8	mRNA cleavage factor complex	CC	1	CC mRNA cleavage factor complex	Gohir.D07G013000.1
GO:0004553	0.00729524	3	368	hydrolase activity, hydrolyzing O-glycosyl compounds	MF	1	MF hydrolase activity, hydrolyzing O-glycosyl compounds	Gohir.D05G028300.1 , Gohir.D08G108700.1 , Gohir.A07G010900.1
GO:0003729	0.01676095	1	12	mRNA binding	MF	1	MF mRNA binding	Gohir.D07G013000.1
GO:0004867	0.02346717	1	10	serine-type endopeptidase inhibitor activity	MF	1	MF serine-type endopeptidase inhibitor activity	Gohir.A11G128400.1
GO:0004869	0.04464726	1	22	cysteine-type endopeptidase inhibitor activity	MF	1	MF cysteine-type endopeptidase inhibitor activity	Gohir.A01G231300.1

Table 4. Grouping of the 2001 unique genes in Jin668 based on PFAM functional domains.

PFAM Accession	# of genes (members)	PFAM Description
PF00067	42	cytochrome p450
PF14368	35	lipid transfer
PF00010	32	helix-loop-helix DNA-binding domain containing protein, expressed; ROOT HAIR DEFECTIVE6
PF00230	29	aquaporin protein, putative, expressed; plasma membrane intrinsic protein 2;8
PF00249	26	MYB family transcription factor, putative, expressed; myb domain protein 14
PF00847	26	AP2 domain containing protein, expressed; ethylene response factor 1
PF00141	22	peroxidase precursor, putative, expressed; Peroxidase family protein
PF14547	20	NA
PF00201	19	UDP-glucoronosyl and UDP-glucosyl transferase domain containing protein, expressed; UDP-Glycosyltransferase superfamily protein
PF00657	17	GDSL-like lipase/acylhydrolase, putative, expressed; GDSL-like Lipase/Acylhydrolase superfamily protein
PF02458	17	transferase family protein, putative, expressed; HXXXD-type acyl-transferase family protein
PF03106	16	WRKY1, expressed; WRKY family transcription factor
PF14009	15	expressed protein
PF00854	15	peptide transporter PTR2, putative, expressed; peptide transporter 2
PF03195	15	DUF260 domain containing protein, putative, expressed; LOB domain-containing protein 4
PF13639	15	zinc finger, C3HC4 type domain containing protein, expressed; brassinosteroid-responsive RING-H2
PF00403	14	heavy metal-associated domain containing protein, expressed; Heavy metal transport/detoxification superfamily protein
PF00407	13	NA

PF02298	12	NA
PF02309	12	OsIAA31 - Auxin-responsive Aux/IAA gene family member, expressed; AUX/IAA transcriptional regulator family protein
PF00170	12	bZIP transcription factor domain containing protein, expressed; Basic-leucine zipper (bZIP) transcription factor family protein
PF01490	11	amino acid transporter, putative, expressed; amino acid permease 6
PF00314	11	thaumatin, putative, expressed; Pathogenesis-related thaumatin superfamily protein
PF14226,PF03171	11	NA
PF01370	10	dihydroflavonol-4-reductase, putative, expressed; NAD(P)-binding Rossmann-fold superfamily protein
PF02362	10	NA
PF01190	10	POEI20 - Pollen Ole e I allergen and extensin family protein precursor, expressed; Pollen Ole e 1 allergen and extensin family protein
PF07714	10	protein kinase family protein, putative, expressed; ACT-like protein tyrosine kinase family protein
PF00046,PF01852	10	homeobox and START domains containing protein, putative, expressed; homeobox-7
PF00069	9	Protein kinase domain containing protein, expressed; PR5-like receptor kinase
PF05142	9	SHI, putative, expressed; SHI-related sequence 7
PF00106	9	oxidoreductase, short chain dehydrogenase/reductase family domain containing family, expressed; protochlorophyllide oxidoreductase A
PF01277	9	oleosin, putative, expressed; Oleosin family protein
PF04043	9	NA
PF12697	9	hydrolase, alpha/beta fold family protein, putative, expressed; alpha/beta-Hydrolases superfamily protein
PF00295	9	polygalacturonase, putative, expressed; Pectin lyase-like superfamily protein
PF02519	8	OsSAUR57 - Auxin-responsive SAUR gene family member, expressed; SAUR-like auxin-responsive protein family
PF00171	8	aldehyde dehydrogenase, putative, expressed; aldehyde dehydrogenase 5F1
PF02704	8	NA
PF02365	8	No apical meristem protein, putative, expressed; NAC (No Apical Meristem) domain transcriptional regulator superfamily protein
PF13561	8	oxidoreductase, short chain dehydrogenase/reductase family protein, putative, expressed; NAD(P)-binding Rossmann-fold superfamily protein
PF08541,PF08392	8	3-ketoacyl-CoA synthase, putative, expressed; 3-ketoacyl-CoA synthase 9
PF08241	7	S-adenosyl-L-methionine-dependent methyltransferases superfamily protein
PF00332	7	glucan endo-1,3-beta-glucosidase precursor, putative, expressed; O-Glycosyl hydrolases family 17 protein
PF01852,PF00046	7	homeobox and START domains containing protein, putative, expressed; homeodomain GLABROUS 2
PF01095	7	pectinesterase, putative, expressed; Pectin lyase-like superfamily protein
PF03330,PF01357	6	expansin precursor, putative, expressed; expansin B1
PF01596	6	caffeoyl-CoA O-methyltransferase, putative, expressed; S-adenosyl-L-methionine-dependent methyltransferases superfamily protein
PF00891,PF08100	6	O-methyltransferase, putative, expressed; O-methyltransferase 1
PF00400	6	suppressor of phythchrome A, putative, expressed; Transducin/WD40 repeat-like superfamily protein

PF01357,PF03330	6	expansin precursor, putative, expressed; expansin 12
PF08100,PF00891	6	O-methyltransferase, putative, expressed; O-methyltransferase 1
PF03171,PF14226	6	gibberellin 20 oxidase 2, putative, expressed; 2-oxoglutarate (2OG) and Fe(II)-dependent oxygenase superfamily protein
PF00083	6	transporter family protein, putative, expressed; polyol/monosaccharide transporter 5
PF00544	6	pectate lyase precursor, putative, expressed; Pectate lyase family protein
PF04852	6	DUF640 domain containing protein, putative, expressed; Protein of unknown function (DUF640)
PF03168	6	harpin-induced protein 1 domain containing protein, expressed; Late embryogenesis abundant (LEA) hydroxyproline-rich glycoprotein family
PF02183,PF00046	6	homeobox associated leucine zipper, putative, expressed; homeobox 51
PF05678	6	VQ domain containing protein, putative, expressed; VQ motif-containing protein
PF07816	6	expressed protein; Protein of unknown function (DUF1645)
PF00892	6	auxin-induced protein 5NG4, putative, expressed; Walls Are Thin 1
PF07526,PF05920	6	homeobox domain containing protein, expressed; BEL1-like homeodomain 1
PF02701	5	dof zinc finger domain containing protein, putative, expressed; DOF zinc finger protein 2
PF02535	5	metal cation transporter, putative, expressed; zinc transporter 10 precursor
PF13193,PF00501	5	AMP-binding enzyme, putative, expressed; acyl-activating enzyme 7
PF15699	5	NA
PF03641	5	uncharacterized protein PA4923, putative, expressed; Putative lysine decarboxylase family protein
PF00241	5	actin-depolymerizing factor, putative, expressed; actin depolymerizing factor 5
PF00248	5	NA
PF04749	5	uncharacterized Cys-rich domain containing protein, putative, expressed; PLANT CADMIUM RESISTANCE 2
PF00561	5	hydrolase, alpha/beta fold family domain containing protein, expressed; esterase/lipase/thioesterase family protein
PF00903	5	glyoxalase family protein, putative, expressed; Lactoylglutathione lyase / glyoxalase I family protein
PF00005,PF01061	5	ABC-2 type transporter domain containing protein, expressed; ABC-2 type transporter family protein
PF07714,PF01657	5	TKL_IRAK_DUF26-lc.20 - DUF26 kinases have homology to DUF26 containing loci, expressed; cysteine-rich RLK (RECEPTOR-like protein kinase) 8
PF14541,PF14543	5	eukaryotic aspartyl protease domain containing protein, expressed; Eukaryotic aspartyl protease family protein
PF00107,PF08240	5	dehydrogenase, putative, expressed; Zinc-binding alcohol dehydrogenase family protein
PF11960,PF00487	5	fatty acid desaturase, putative, expressed; fatty acid desaturase 2
PF04640	5	zinc-binding protein, putative, expressed; PLATZ transcription factor family protein
PF00501,PF13193	4	AMP-binding domain containing protein, expressed; OPC-8:0 CoA ligase1
PF05097	4	NA
PF04185	4	phosphoesterase family protein, putative, expressed; non-specific phospholipase C1
PF01112	4	L-asparaginase precursor protein, putative, expressed; N-terminal nucleophile aminohydrolases (Ntn hydrolases) superfamily protein
PF06404	4	phytosulfokines precursor, putative, expressed; PHYTOSULFOKINE 3 PRECURSOR
PF04398	4	expressed protein; Protein of unknown function, DUF538
PF00504	4	chlorophyll A-B binding protein, putative, expressed; light-harvesting chlorophyll B-binding protein 3

PF00808	4	core histone H2A/H2B/H3/H4, putative, expressed; nuclear factor Y, subunit C1
PF08137	4	NA
PF02045	4	nuclear transcription factor Y subunit, putative, expressed; nuclear factor Y, subunit A9
PF00664,PF00005	4	ABC transporter, ATP-binding protein, putative, expressed; multidrug resistance-associated protein 14
PF00011	4	heat shock protein, putative, expressed; HSP20-like chaperones superfamily protein
PF00646	4	OsFBL19 - F-box domain and LRR containing protein, expressed; VIER F-box proteine 3
PF08240,PF00107	4	dehydrogenase, putative, expressed; alcohol dehydrogenase 1
PF00046,PF02183	4	homeobox associated leucine zipper, putative, expressed; homeobox-leucine zipper protein 17
PF00190	4	NA
PF03006	4	haemolysin-III, putative, expressed; heptahelical protein 4
PF04832	4	SOUL heme-binding protein, putative, expressed; SOUL heme-binding family protein
PF16845	4	cysteine proteinase inhibitor 8 precursor, putative, expressed; Cystatin/monellin superfamily protein
PF14543,PF14541	4	NA
PF04535	4	membrane associated DUF588 domain containing protein, putative, expressed; Uncharacterised protein family (UPF0497)
PF01554	4	MATE efflux family protein, putative, expressed; MATE efflux family protein
PF00450	4	OsSCP48 - Putative Serine Carboxypeptidase homologue, expressed; serine carboxypeptidase-like 44
PF07983	4	X8 domain containing protein, expressed; Carbohydrate-binding X8 domain superfamily protein
PF00155	4	aminotransferase, classes I and II, domain containing protein, expressed; alanine aminotransferas
PF00046	4	homeobox associated leucine zipper, putative, expressed; homeobox-leucine zipper protein 17
PF00111	4	2Fe-2S iron-sulfur cluster binding domain containing protein, expressed; ferredoxin 1
PF00335	4	tetraspanin family protein, putative, expressed; Tetraspanin family protein
PF05553,PF14364	3	NA
PF00487,PF11960	3	fatty acid desaturase, putative, expressed; fatty acid desaturase 2
PF01627	3	histidine-containing phosphotransfer protein, putative, expressed; HPT phosphotransmitter 4
PF05212	3	lysine ketoglutarate reductase trans-splicing related 1, putative, expressed; Protein of unknown function (DUF707)
PF03552	3	CSLE6 - cellulose synthase-like family E, expressed; cellulose synthase like E1
PF08417,PF00355	3	chlorophyllide a oxygenase, chloroplast precursor, putative, expressed; Pheophorbide a oxygenase family protein with Rieske [2Fe-2S] domain
PF00320	3	expressed protein; GATA transcription factor 15
PF00005	3	white-brown complex homolog protein, putative, expressed; ABC transporter family protein
PF04862	3	expressed protein; Protein of unknown function, DUF642
PF01439	3	metallothionein, putative, expressed; metallothionein 2A
PF04770	3	ZF-HD protein dimerisation region containing protein, expressed; homeobox protein 24
PF00743	3	NA
PF05617	3	NA
PF03790,PF03791, PF05920	3	homeobox protein knotted-1, putative, expressed; KNOTTED-like homeobox of Arabidopsis thaliana 7
PF12076,PF04116	3	WAX2, putative, expressed; Fatty acid hydroxylase superfamily

PF05899	3	enzyme of the cupin superfamily protein, putative, expressed; RmlC-like cupins superfamily protein
PF03000	3	BTBN13 - Bric-a-Brac, Tramtrack, Broad Complex BTB domain with non-phototropic hypocotyl 3 NPH3 and coiled-coil domains, expressed; Phototropic-responsive NPH3 family protein
PF00685	3	NA
PF06884	3	DUF1264 domain containing protein, putative, expressed; Protein of unknown function (DUF1264)
PF12906	3	zinc finger, C3HC4 type, domain containing protein, expressed; RING/U-box superfamily protein
PF08392,PF08541	3	3-ketoacyl-CoA synthase precursor, putative, expressed; 3-ketoacyl-CoA synthase 19
PF03080,PF14365	3	carboxyl-terminal peptidase, putative, expressed; Protein of Unknown Function (DUF239)
PF05498	3	NA
PF01095,PF04043	3	pectinesterase, putative, expressed; Plant invertase/pectin methylesterase inhibitor superfamily
PF05911	3	plant protein of unknown function DUF869 domain containing protein, expressed; Plant protein of unknown function (DUF869)
PF00481	3	protein phosphatase 2C, putative, expressed; Protein phosphatase 2C family protein
PF03083	3	nodulin MtN3 family protein, putative, expressed; senescence-associated gene 29
PF02042	3	RWP-RK domain-containing protein, putative, expressed; RWP-RK domain-containing protein
PF14310,PF00933, PF01915	3	beta-D-xylosidase, putative, expressed; beta-D-xylosidase 4
PF03479	3	DNA binding protein, putative, expressed; AT-hook motif nuclear-localized protein 20
PF00168	3	NA
PF14144	3	expressed protein
PF00967	3	WIP3 - Wound-induced protein precursor, expressed; pathogenesis-related 4
PF00234	3	NA
PF05755	3	stress-related protein, putative, expressed; Rubber elongation factor protein (REF)
PF04570	3	NA
PF00188	3	SCP-like extracellular protein, expressed; pathogenesis-related protein-1-like
PF00445	3	NA
PF06955,PF00722	3	glycosyl hydrolases family 16, putative, expressed; xyloglucan:xyloglucosyl transferase 33
PF04844	3	DUF623 domain containing protein, expressed; ovate family protein 6
PF00125	2	histone H3, putative, expressed; Histone superfamily protein
PF09265,PF01565	2	cytokinin dehydrogenase precursor, putative, expressed; cytokinin oxidase 5
PF00082,PF05922	2	OsSub27 - Putative Subtilisin homologue, expressed; Subtilisin-like serine endopeptidase family protein
PF02798,PF13410	2	glutathione S-transferase, putative, expressed; glutathione S-transferase tau 7
PF02068	2	NA
PF03790,PF03791, PF05920,PF03789	2	Homeobox domain containing protein, expressed; KNOTTED-like from Arabidopsis thaliana
PF14705	2	expressed protein
PF00955	2	boron transporter protein, putative, expressed; HCO3- transporter family
PF00982,PF02358	2	trehalose synthase, putative, expressed; UDP-Glycosyltransferase / trehalose-phosphatase family protein

PF03140	2	NA
PF01301,PF02140	2	beta-galactosidase precursor, putative, expressed; glycosyl hydrolase family 35 protein
PF13855	2	leucine-rich repeat-containing protein 40, putative, expressed; Leucine-rich repeat (LRR) family protein
PF06136	2	domain of unknown function DUF966 domain containing protein, expressed; Domain of unknown function (DUF966)
PF00079	2	serpin domain containing protein, putative, expressed; Serine protease inhibitor (SERPIN) family protein
PF05920,PF07526	2	homeodomain protein, putative, expressed; POX (plant homeobox) family protein
PF03999	2	microtubule associated protein, putative, expressed; microtubule-associated proteins 65-1
PF01699	2	sodium/calcium exchanger protein, putative, expressed; cation exchanger 5
PF03016	2	exostosin family domain containing protein, expressed; Exostosin family protein
PF07714,PF08263, PF13855	2	inactive receptor kinase At2g26730 precursor, putative, expressed; Leucine-rich repeat protein kinase family protein
PF07011	2	EARLY flowering protein, putative, expressed; ELF4-like 3
PF03514	2	gibberellin response modulator protein, putative, expressed; GRAS family transcription factor
PF01344	2	OsFBK15 - F-box domain and kelch repeat containing protein, expressed; Galactose oxidase/kelch repeat superfamily protein
PF03912	2	photosystem II reaction center PSB28 protein, chloroplast precursor, putative, expressed; photosystem II reaction center PSB28 protein
PF04864	2	alliin lyase precursor, putative, expressed; tryptophan aminotransferase of Arabidopsis 1
PF10604	2	cyclase/dehydrase family protein, putative, expressed; PYR1-like 4
PF05564	2	auxin-repressed protein, putative, expressed; Dormancy/auxin associated family protein
PF16211,PF00125	2	core histone H2A/H2B/H3/H4, putative, expressed; histone H2A 10
PF03372	2	NA
PF01715	2	IPP transferase, putative, expressed; isopentenyltransferase 9
PF00514	2	armadillo/beta-catenin repeat family protein, putative, expressed; armadillo repeat only 1
PF00759	2	endoglucanase, putative, expressed; glycosyl hydrolase 9B8
PF01221	2	dynein light chain type 1 domain containing protein, expressed; Dynein light chain type 1 family protein
PF00046,PF04618, PF02183	2	homeobox associated leucine zipper, putative, expressed; homeobox from Arabidopsis thaliana
PF16913	2	purine permease, putative, expressed; purine permease 3
PF02784	2	pyridoxal-dependent decarboxylase protein, putative, expressed; arginine decarboxylase 2
PF02362,PF07496	2	B3 DNA binding domain containing protein, putative, expressed; HSI2-like 1
PF00255	2	glutathione peroxidase, putative, expressed; glutathione peroxidase 1
PF00072,PF00249	2	two-component response regulator, putative, expressed; response regulator 1
PF01918	2	protein of unknown function domain containing protein, expressed; Alba DNA/RNA-binding protein
PF12796,PF13962	2	cadmium tolerance factor, putative, expressed; Ankyrin repeat family protein
PF03765,PF00650	2	CRAL/TRIO domain containing protein, expressed; Sec14p-like phosphatidylinositol transfer family protein
PF04043,PF01095	2	pectinesterase, putative, expressed; Plant invertase/pectin methylesterase inhibitor superfamily
PF08212	2	OsCHL Chloroplastic lipocalin, expressed; chloroplastic lipocalin

PF04783,PF04782	2	NA
PF07911	2	expressed protein; Protein of unknown function (DUF1677)
PF03181	2	BURP domain containing protein, expressed; polygalacturonase 2
PF00109,PF02801	2	3-oxoacyl-synthase, putative, expressed; fatty acid biosynthesis 1
PF13347	2	sucrose transporter, putativ, expressed; sucrose-proton symporter 2
PF01501	2	glycosyl transferase family 8, putative, expressed; galacturonosyltransferase 3
PF00582	2	universal stress protein domain containing protein, putative, expressed; Adenine nucleotide alpha hydrolases-like superfamily protein
PF12481	2	stem-specific protein TSJT1, putative, expressed; Aluminium induced protein with YGL and LRDR motifs
PF00583	2	acetyltransferase, GNAT family, putative, expressed; glucose-6-phosphate acetyltransferase 1
PF13713,PF08381	2	Disease resistance/zinc finger/chromosome condensation-like region protein, putative, expressed; BREVIS RADIX-like 4
PF05910	2	expressed protein; Plant protein of unknown function (DUF868)
PF01740,PF00916	2	sulfate transporter, putative, expressed; sulfate transporter 91
PF01061,PF00005	2	ABC-2 type transporter domain containing protein, expressed; ABC-2 type transporter family protein
PF04674	2	phosphate-induced protein 1 conserved region domain containing protein, expressed; Phosphate-responsive 1 family protein
PF03634	2	TCP family transcription factor, putative, expressed; TCP family transcription factor 4
PF06203,PF00643	2	NA
PF06943	2	TFIIH basal transcription factor complex p52 subunit, putative, expressed; transcription factor-related
PF00891	2	O-methyltransferase, putative, expressed; O-methyltransferase family protein
PF00319,PF01486	2	OsMADS4 - MADS-box family gene with MIKCc type-box, expressed; K-box region and MADS-box transcription factor family protein
PF06094	2	AIG2-like family domain containing protein, expressed; AIG2-like (avirulence induced gene) family protein
PF10714	2	NA
PF08031,PF01565	2	berberine and berberine like domain containing protein, expressed; FAD-binding Berberine family protein
PF07732,PF00394	2	NA
PF08802,PF00355	2	cytochrome b6-f complex iron-sulfur subunit, chloroplast precursor, putative, expressed; photosynthetic electron transfer C
PF05340	2	expressed protein
PF02450	2	lecithin cholesterol acyltransferase, putative, expressed; alpha/beta-Hydrolases superfamily protein
PF03097	2	expressed protein; Endosomal targeting BRO1-like domain-containing protein
PF00394,PF07731, PF07732	2	monocopper oxidase, putative, expressed; SKU5 similar 5
PF14215,PF00010	2	transcription factor MYC7E, putative, expressed; Basic helix-loop-helix (bHLH) DNA-binding family protein
PF13499,PF00036	2	OsCML17 - Calmodulin-related calcium sensor protein, expressed; Calcium-binding EF-hand family protein
PF00168,PF08372	2	anthranilate phosphoribosyltransferase, putative, expressed; Calcium-dependent lipid-binding (CaLB domain) plant phosphoribosyltransferase family protein
PF00072	2	two-component response regulator, putative, expressed; response regulator 2

PF03088	2	strictosidine synthase, putative, expressed; strictosidine synthase-like 2
PF00732,PF05199	2	HOTHEAD precursor, putative, expressed; Glucose-methanol-choline (GMC) oxidoreductase family protein
PF05055	2	DUF677 domain containing protein, putative, expressed; Protein of unknown function (DUF677)
PF14159	2	expressed protein
PF00182	2	CHIT8 - Chitinase family protein precursor, expressed; basic chitinase
PF11493	2	expressed protein
PF03767	2	HAD superfamily phosphatase, putative, expressed; HAD superfamily, subfamily IIIB acid phosphatase
PF13602,PF08240	2	dehydrogenase, putative, expressed; GroES-like zinc-binding alcohol dehydrogenase family protein
PF07687,PF01546	2	hydrolase, putative, expressed; Peptidase M20/M25/M40 family protein
PF03789,PF05920, PF03790,PF03791	2	Homeobox domain containing protein, expressed; KNOX/ELK homeobox transcription factor
PF03321	2	OsGH3.3 - Probable indole-3-acetic acid-amido synthetase, expressed; Auxin-responsive GH3 family protein
PF01536	2	adenosylmethionine decarboxylase, putative, expressed; Adenosylmethionine decarboxylase family protein
PF00332,PF07983	2	glucan endo-1,3-beta-glucosidase precursor, putative, expressed; O-Glycosyl hydrolases family 17 protein
PF00319	2	NA
PF04542,PF04545, PF04539	2	RNA polymerase sigma factor, putative, expressed; RNApolymerase sigma-subunit C
PF00156	2	phosphoribosyl transferase, putative, expressed; adenine phosphoribosyltransferase 5
PF00293	2	hydrolase, NUDIX family, domain containing protein, expressed; nudix hydrolase homolog 2
PF00240	2	NA
PF03478,PF00646	2	NA
PF00210	2	ferritin-1, chloroplast precursor, putative, expressed; ferretin 1
PF03822,PF00069	2	CAMK_KIN1/SNF1/Nim1_like.17 - CAMK includes calcium/calmodulin depedent protein kinases, expressed; CBL-interacting protein kinase 7
PF04725	2	photosystem II 10 kDa polypeptide, chloroplast precursor, putative, expressed; photosystem II subunit R
PF03547	2	auxin efflux carrier component, putative, expressed; Auxin efflux carrier family protein
PF12609	2	Wound-responsive family protein
PF12023	2	NA
PF00651,PF03000	2	BTBN6 - Bric-a-Brac, Tramtrack, Broad Complex BTB domain with non-phototropic hypocotyl 3 NPH3 and coiled-coil domains, expressed; Phototropic-responsive NPH3 family protein
PF13905,PF03107	2	protein disulfide isomerase, putative, expressed; protein kinase C-like zinc finger protein
PF06376	2	NA
PF00704	2	NA
PF00085	2	thioredoxin, putative, expressed; C-terminal cysteine residue is changed to a serine 1
PF03094	2	MLO domain containing protein, putative, expressed; Seven transmembrane MLO family protein
PF00122,PF00690, PF00702	2	plasma membrane ATPase, putative, expressed; H(+)-ATPase 9
PF00257	2	NA

PF13921	2	NA
PF00005,PF00664	2	multidrug resistance protein, putative, expressed; ABC transporter family protein
PF13912	2	ZOS7-01 - C2H2 zinc finger protein, expressed; C2H2 and C2HC zinc fingers superfamily protein
PF06813	2	nodulin, putative, expressed; Major facilitator superfamily protein
PF01106	2	nifU, putative, expressed; NIFU-like protein 2
PF00916,PF01740	2	sulfate transporter, putative, expressed; sulfate transporter 1;3
PF00571	2	cystathionin beta synthase protein, putative, expressed; CBS domain-containing protein
PF00125,PF16211	2	core histone H2A/H2B/H3/H4, putative, expressed; histone H2A 10
PF07123	2	photosystem II reaction center W protein, chloroplast precursor, putative, expressed; photosystem II reaction center W
PF03330	2	beta-expansin precursor, putative, expressed; plant natriuretic peptide A
PF04526,PF03188	2	NA
PF00581	2	rhodanese-like, putative, expressed; Rhodanese/Cell cycle control phosphatase superfamily protein
PF12796,PF07714	2	serine/threonine protein kinase, putative, expressed; Integrin-linked protein kinase family
PF13417,PF00043	2	glutathione S-transferase, putative, expressed; glutathione S-transferase tau 7
PF14144,PF00170	2	transcription factor, putative, expressed; bZIP transcription factor family protein
PF00175,PF00258, PF00667	2	NADPH reductase, putative, expressed; P450 reductase 1
PF02728,PF02727, PF01179	2	copper methylamine oxidase precursor, putative, expressed; Copper amine oxidase family protein
PF02453	2	reticulon domain containing protein, putative, expressed; Reticulon family protein
PF04450	1	secretory protein, putative, expressed; Plant basic secretory protein (BSP) family protein
PF00902	1	uncharacterized tatC-like protein ymf16, putative, expressed; Sec-independent periplasmic protein translocase
PF07859	1	CXE carboxylesterase, putative, expressed; carboxyesterase 16
PF03386	1	NA
PF01061,PF00005, PF08370	1	pleiotropic drug resistance protein, putative, expressed; pleiotropic drug resistance 10
PF07002,PF13920	1	copine, putative, expressed; RING domain ligase2
PF03018	1	dirigent, putative, expressed; Disease resistance-responsive (dirigent-like protein) family protein
PF00724	1	12-oxophytodienoate reductase, putative, expressed; 12-oxophytodienoate reductase 2
PF05694	1	selenium-binding protein, putative, expressed; selenium-binding protein 2
PF01679	1	OsRCI2-11 - Hydrophobic protein OSR8, expressed; Low temperature and salt responsive protein family
PF14309,PF14383	1	NA
PF08523,PF01381	1	endothelial differentiation-related factor 1, putative, expressed; multiprotein bridging factor 1A
PF13499	1	NA
PF02358	1	uncharacterized glycosyl hydrolase Rv2006/MT2062, putative, expressed; Haloacid dehalogenase-like hydrolase (HAD) superfamily protein
PF01429	1	NA
PF09713	1	plant-specific domain TIGR01589 family protein, expressed; Plant protein 1589 of unknown function

PF01564,PF17284	1	spermidine synthase, putative, expressed; S-adenosyl-L-methionine-dependent methyltransferases superfamily protein
PF00043,PF02798	1	glutathione S-transferase, putative, expressed; glutathione S-transferase tau 7
PF08491	1	squalene monooxygenase, putative, expressed; FAD/NAD(P)-binding oxidoreductase family protein
PF08879,PF08880	1	NA
PF12796,PF13962, PF00023	1	ankyrin repeat-containing protein, putative, expressed; Ankyrin repeat family protein
PF12515,PF00689, PF00122,PF00690, PF00702	1	calcium-transporting ATPase, plasma membrane-type, putative, expressed; autoinhibited Ca(2+)-ATPase 9
PF04720	1	plant-specific domain TIGR01615 family protein, expressed; Protein of unknown function (DUF506)
PF00149	1	Ser/Thr protein phosphatase family protein, putative, expressed; Calcineurin-like metallo-phosphoesterase superfamily protein
PF08268	1	NA
PF04734,PF17048	1	neutral ceramidase precursor, putative, expressed; Neutral/alkaline non-lysosomal ceramidase
PF12710,PF01553	1	glycerol-3-phosphate acyltransferase, putative, expressed; glycerol-3-phosphate acyltransferase 6
PF13540,PF00069	1	TKL_IRAK_CR4L.7 - The CR4L subfamily has homology with Crinkly4, expressed; CRINKLY4 related 4
PF01477	1	wound/stress protein, putative, expressed; Lipase/lipooxygenase, PLAT/LH2 family protein
PF13178,PF00612	1	NA
PF00069,PF13947, PF07645	1	OsWAK13 - OsWAK receptor-like protein kinase, expressed; wall-associated kinase 2
PF13855,PF11721	1	NA
PF00628,PF01426	1	ES43 protein, putative, expressed; PHD finger family protein / bromo-adjacent homology (BAH) domain-containing protein
PF02353	1	Cyclopropane-fatty-acyl-phospholipid synthase, putative, expressed; Cyclopropane-fatty-acyl-phospholipid synthase
PF01145	1	hypersensitive-induced response protein, putative, expressed; SPFH/Band 7/PHB domain-containing membrane-associated protein family
PF03649	1	ABC transporter, membrane-spanning/permease subunit, putative, expressed; aluminum sensitive 3
PF04654	1	expressed protein; Protein of unknown function, DUF599
PF00266	1	aminotransferase, putative, expressed; alanine:glyoxylate aminotransferase
PF00702,PF13246, PF00690,PF00689, PF00122	1	calcium-transporting ATPase, endoplasmic reticulum-type, putative, expressed; ER-type Ca2+-ATPase 1
PF05705	1	expressed protein; Protein of unknown function DUF829, transmembrane 53
PF03127,PF00790	1	VHS and GAT domain containing protein, expressed; ENTH/VHS/GAT family protein
PF14380,PF13947, PF00069	1	OsWAK1 - OsWAK receptor-like cytoplasmic kinase OsWAK-RLCK, expressed; Protein kinase superfamily protein
PF10237	1	N2, N2-dimethylguanosine tRNA methyltransferase, putative, expressed; nucleic acid binding;methyltransferases

PF16891,PF00149	1	Ser/Thr protein phosphatase family protein, putative, expressed; Calcineurin-like metallo-phosphoesterase superfamily protein
PF00686	1	NA
PF03081	1	ATEXO70C2, putative, expressed; exocyst subunit exo70 family protein C1
PF03470,PF03469, PF03468	1	transcription factor X1, putative, expressed; XH/XS domain-containing protein
PF01161	1	phosphatidylethanolamine-binding protein, putative, expressed; PEBP (phosphatidylethanolamine-binding protein) family protein
PF01063	1	aminotransferase domain containing protein, putative, expressed; D-aminoacid aminotransferase-like PLP-dependent enzymes superfamily protein
PF00069,PF03822	1	CAMK_KIN1/SNF1/Nim1_like.25 - CAMK includes calcium/calmodulin depedent protein kinases, expressed; serine/threonine protein kinase 1
PF01293	1	phosphoenolpyruvate carboxykinase, putative, expressed; phosphoenolpyruvate carboxykinase 1
PF08387	1	NA
PF03134	1	HVA22, putative, expressed; HVA22-like protein J
PF08240,PF13602	1	dehydrogenase, putative, expressed; Oxidoreductase, zinc-binding dehydrogenase family protein
PF00388	1	ki1 protein, putative, expressed; PLC-like phosphodiesterases superfamily protein
PF02778	1	tRNA-splicing endonuclease subunit Sen2, putative, expressed; splicing endonuclease 1
PF02151,PF04379	1	NA
PF13768	1	von Willebrand factor type A domain containing protein, expressed; inter-alpha-trypsin inhibitor heavy chain-related
PF11998	1	photosystem-II repair protein, putative, expressed
PF07839	1	NA
PF07645,PF13947	1	NA
PF08244,PF00251	1	glycosyl hydrolases, putative, expressed; Glycosyl hydrolases family 32 protein
PF14389,PF00169, PF00620	1	pleckstrin homology domain-containing protein, putative, expressed; Rho GTPase activation protein (RhoGAP) with PH domain
PF04078	1	expressed protein; Cell differentiation, Rcd1-like protein
PF05922	1	NA
PF15054	1	expressed protein
PF00494	1	squalene synthetase, putative, expressed; squalene synthase 1
PF01117,PF07468	1	NA
PF04588	1	hypoxia-responsive family protein, putative, expressed; Hypoxia-responsive family protein
PF00122,PF00689	1	NA
PF13833	1	OsCML11 - Calmodulin-related calcium sensor protein, expressed; Calcium-binding EF-hand family protein
PF03107	1	NA
PF08132	1	S-adenosyl-l-methionine decarboxylase leader peptide, putative, expressed; conserved peptide upstream open reading frame 10
PF11820	1	expressed protein; Protein of unknown function (DUF 3339)

PF12796,PF00023	1	ankyrin repeat domain-containing protein 28, putative, expressed; XB3 ortholog 1 in Arabidopsis thaliana
PF00805	1	thylakoid lumenal protein, putative, expressed; Tetratricopeptide repeat (TPR)-like superfamily protein
PF00108,PF02803	1	acetyl-CoA acetyltransferase, cytosolic, putative, expressed; Thiolase family protein
PF01301	1	beta-galactosidase precursor, putative, expressed; beta-galactosidase 7
PF01657	1	cysteine-rich repeat secretory protein precursor, putative, expressed; plasmodesmata-located protein 6
PF00195,PF02797	1	chalcone synthase, putative, expressed; Chalcone and stilbene synthase family protein
PF07748,PF01074, PF09261	1	lysosomal alpha-mannosidase precursor, putative, expressed; Glycosyl hydrolase family 38 protein
PF05907	1	GA18326-PA, putative, expressed
PF08263,PF13855, PF07714	1	LRR receptor-like protein kinase, putative, expressed; Leucine-rich repeat protein kinase family protein
PF14416,PF13839	1	PMR5, putative, expressed; TRICHOME BIREFRINGENCE-LIKE 8
PF12643	1	MazG nucleotide pyrophosphohydrolase domain containing protein, expressed
PF00278,PF02784	1	NA
PF02800,PF00044	1	glyceraldehyde-3-phosphate dehydrogenase, putative, expressed; glyceraldehyde-3-phosphate dehydrogenase C2
PF01593,PF02353	1	Cyclopropane-fatty-acyl-phospholipid synthase, putative, expressed; Cyclopropane-fatty-acyl-phospholipid synthase
PF08513	1	F-box-like/WD repeat-containing protein TBL1XR1-A, putative, expressed; WD-40 repeat family protein
PF00096	1	ZOS2-09 - C2H2 zinc finger protein, expressed; zinc finger (C2H2 type) family protein
PF06172	1	cupin superfamily protein, putative, expressed
PF00646,PF08387	1	NA
PF03763,PF03766	1	NA
PF08022,PF08414, PF01794,PF08030	1	ferric reductase, putative, expressed; Riboflavin synthase-like superfamily protein
PF04864,PF04863	1	alliin lyase precursor, putative, expressed; Pyridoxal phosphate (PLP)-dependent transferases superfamily protein
PF00702,PF00690, PF00122	1	plasma membrane ATPase, putative, expressed; H(+)-ATPase 1
PF03951,PF00120	1	glutamine synthetase, catalytic domain containing protein, expressed; glutamine synthase clone R1
PF13606,PF13962, PF12796	1	NA
PF13920	1	NA
PF01135	1	protein-L-isoaspartate O-methyltransferase, putative, expressed; protein-l-isoaspartate methyltransferase 1
PF00076	1	RNA recognition motif containing protein, putative, expressed; RNA binding (RRM/RBD/RNP motifs) family protein
PF00076,PF00658	1	polyadenylate-binding protein, putative, expressed; poly(A) binding protein 2
PF02178,PF00538	1	NA
PF13812,PF13041	1	pentatricopeptide, putative, expressed; Tetratricopeptide repeat (TPR)-like superfamily protein
PF05637	1	glycosyltransferase, putative, expressed; UDP-xylosyltransferase 2
PF03759	1	ATROPGEF7/ROPGEF7, putative, expressed; rho guanyl-nucleotide exchange factor 1
PF00232	1	Os1bglu1 - beta-mannosidase/glucosidase homologue, expressed; B-S glucosidase 44

PF16884,PF00107	1	NADP-dependent oxidoreductase, putative, expressed; Zinc-binding dehydrogenase family protein
PF03763	1	remorin family protein, putative, expressed; Remorin family protein
PF12937	1	OsFBX330 - F-box domain containing protein, expressed; F-box/RNI-like superfamily protein
PF04554,PF01190	1	NA
PF00538	1	NA
PF13855,PF00069, PF08263	1	NA
PF14363,PF00004	1	mitochondrial chaperone BCS1, putative, expressed; P-loop containing nucleoside triphosphate hydrolases superfamily protein
PF00933,PF14310, PF01915	1	glycosyl hydrolase family 3 protein, putative, expressed; Glycosyl hydrolase family protein
PF01786	1	immutans protein, putative, expressed; alternative oxidase 2
PF01965	1	DJ-1 family protein, putative, expressed; Class I glutamine amidotransferase-like superfamily protein
PF13499,PF00069	1	CAMK_CAMK_like.33 - CAMK includes calcium/calmodulin depedent protein kinases, expressed; calmodulin-domain protein kinase 9
PF03357	1	SNF7 domain containing protein, putative, expressed; SNF7 family protein
PF03476,PF03473	1	MOSC domain-containing protein, mitochondrial precursor, putative, expressed; Molybdenum cofactor sulfurase family protein
PF02785,PF02786, PF00289	1	methylcrotonoyl-CoA carboxylase subunit alpha, mitochondrial precursor, putative, expressed; acetyl Co-enzyme a carboxylase biotin carboxylase subunit
PF00304	1	DEF8 - Defensin and Defensin-like DEFL family, expressed; Scorpion toxin-like knottin superfamily protein
PF03059	1	nicotianamine synthase, putative, expressed; nicotianamine synthase 3
PF05922,PF02225, PF00082	1	OsSub30 - Putative Subtilisin homologue, expressed; Subtilase family protein
PF04618,PF02183, PF00046	1	homeobox associated leucine zipper, putative, expressed; homeobox protein 2
PF00191	1	annexin, putative, expressed; annexin 2
PF05542	1	seed maturation protein PM23, putative, expressed
PF00487	1	fatty acid desaturase 5
PF02797,PF00195	1	chalcone synthase, putative, expressed; Chalcone and stilbene synthase family protein
PF03106,PF10533	1	WRKY68, expressed; WRKY DNA-binding protein 15
PF00538,PF02178	1	NA
PF00182,PF00187	1	CHIT8 - Chitinase family protein precursor, expressed; basic chitinase
PF08787	1	NA
PF03939,PF00276	1	60S ribosomal protein L23A, putative, expressed; ribosomal protein L23AB
PF03242	1	NA
PF13520,PF13906	1	amino acid permease family protein, putative, expressed; cationic amino acid transporter 7
PF04134	1	thiol-disulphide oxidoreductase DCC, putative, expressed; Putative thiol-disulphide oxidoreductase DCC

PF05349	1	expressed protein
PF00004,PF01434	1	OsFtsH2 FtsH protease, homologue of AtFtsH2/8, expressed; FtsH extracellular protease family
PF02803,PF00108	1	3-ketoacyl-CoA thiolase, peroxisomal precursor, putative, expressed; peroxisomal 3-ketoacyl-CoA thiolase 3
PF04669	1	plant-specific domain TIGR01627 family protein, expressed; Protein of unknown function (DUF579)
PF06507,PF02309, PF02362	1	auxin response factor, putative, expressed; auxin response factor 6
PF06232	1	embryo-specific 3, putative, expressed; Embryo-specific protein 3, (ATS3)
PF00107	1	NADP-dependent oxidoreductase, putative, expressed; Zinc-binding dehydrogenase family protein
PF00226	1	NA
PF00696	1	amino acid kinase, putative, expressed; delta1-pyrroline-5-carboxylate synthase 1
PF00187,PF00182	1	CHIT8 - Chitinase family protein precursor, expressed; basic chitinase
PF00004,PF05362, PF02190	1	OsLonP4 - Putative Lon protease homologue, expressed; lon protease 2
PF00658,PF00076	1	polyadenylate-binding protein, putative, expressed; poly(A) binding protein 2
PF01477,PF00305	1	lipoxygenase, putative, expressed; PLAT/LH2 domain-containing lipoxygenase family protein
PF00689,PF00702	1	calcium-transporting ATPase, plasma membrane-type, putative, expressed; ATPase E1-E2 type family protein / haloacid dehalogenase-like hydrolase family protein
PF00071	1	ras-related protein, putative, expressed; Ras-related small GTP-binding family protein
PF14365,PF03080	1	carboxyl-terminal peptidase, putative, expressed; Protein of Unknown Function (DUF239)
PF03953,PF00091	1	tubulin/FtsZ domain containing protein, putative, expressed; tubulin beta-1 chain
PF06966	1	DUF1295 domain containing protein, putative, expressed; Protein of unknown function (DUF1295)
PF17048,PF04734	1	neutral ceramidase precursor, putative, expressed; Neutral/alkaline non-lysosomal ceramidase
PF10998	1	integral membrane protein, putative, expressed
PF00127	1	plastocyanin, chloroplast precursor, putative, expressed; Cupredoxin superfamily protein
PF05922,PF00082	1	OsSub59 - Putative Subtilisin homologue, expressed; Subtilase family protein
PF12056	1	extracellular ligand-gated ion channel, putative, expressed; Protein of unknown function (DUF3537)
PF01694	1	OsRhmbd15 - Putative Rhomboid homologue, expressed; RHOMBOID-like protein 10
PF13589	1	ATP-binding region, ATPase-like domain containing protein, expressed; Histidine kinase-, DNA gyrase B-, and HSP90-like ATPase family protein
PF17181	1	NA
PF00646,PF13516	1	OsFBL22 - F-box domain and LRR containing protein, expressed; RNI-like superfamily protein
PF00043	1	glutathione S-transferase, putative, expressed; glutathione S-transferase PHI 9
PF00578,PF10417	1	peroxiredoxin, putative, expressed; 1-cysteine peroxiredoxin 1
PF14364,PF05553	1	fiber expressed protein, putative, expressed; Protein of unknown function (DUF761)
PF13962,PF12796, PF13606	1	NA
PF03188	1	cytochrome b561, putative, expressed; Cytochrome b561/ferric reductase transmembrane protein family

PF16653,PF04455, PF03435,PF05222	1	saccharopine dehydrogenase, putative, expressed; lysine-ketoglutarate reductase/saccharopine dehydrogenase bifunctional enzyme
PF06549	1	NA
PF08387,PF07723, PF00646	1	NA
PF13516	1	NA
PF16863,PF01055, PF13802	1	glycosyl hydrolase, family 31, putative, expressed; Glycosyl hydrolases family 31 protein
PF13417	1	glutathione S-transferase, N-terminal domain containing protein, expressed; Thioredoxin family protein
PF00168,PF12357, PF13091,PF00614	1	phospholipase D, putative, expressed; phospholipase D delta
PF00268	1	ribonucleoside-diphosphate reductase small chain, putative, expressed; ribonucleotide reductase 2A
PF01061,PF06422	1	ABC-2 type transporter domain containing protein, expressed; ABC-2 type transporter family protein
PF05553	1	NA
PF00010,PF14215	1	NA
PF02485	1	xylosyltransferase, putative, expressed; Core-2/I-branching beta-1,6-N-acetylglucosaminyltransferase family protein
PF00698	1	malonyl CoA-acyl carrier protein transacylase, mitochondrial precursor, putative, expressed; catalytics;transferases;[acyl-carrier-protein] S-malonyltransferases;binding
PF01657,PF07714	1	TKL_IRAK_DUF26-lf.2 - DUF26 kinases have homology to DUF26 containing loci, expressed; cysteine-rich RLK (RECEPTOR-like protein kinase) 2
PF03171	1	1-aminocyclopropane-1-carboxylate oxidase homolog 2, putative, expressed; 2-oxoglutarate (2OG) and Fe(II)-dependent oxygenase superfamily protein
PF05691	1	uncharacterized glycosyltransferase, putative, expressed; seed imbibition 2
PF04059	1	NA
PF01266	1	FAD dependent oxidoreductase domain containing protein, expressed; FAD-dependent oxidoreductase family protein
PF06624	1	membrane protein, putative, expressed; Ribosome associated membrane protein RAMP4
PF00650,PF03765	1	SEC14 cytosolic factor family protein, putative, expressed; Sec14p-like phosphatidylinositol transfer family protein
PF00046,PF01852, PF08670	1	START domain containing protein, expressed; Homeobox-leucine zipper family protein / lipid-binding START domain-containing protein
PF06596	1	ultraviolet-B-repressible protein, putative, expressed; photosystem II subunit X
PF08449	1	transporter family protein, putative, expressed; UDP-N-acetylglucosamine (UAA) transporter family
PF06507,PF02362	1	auxin response factor 18, putative, expressed; auxin response factor 10
PF12799	1	protein phosphatase 1 regulatory subunit SDS22, putative, expressed; Leucine-rich repeat (LRR) family protein
PF15511	1	Core histone H2A/H2B/H3/H4 domain containing protein, putative, expressed; histone H4
PF02469	1	fasciclin-like arabinogalactan protein 8 precursor, putative, expressed; FASCICLIN-like arabinogalactan 1
PF00924	1	uncharacterized mscS family protein, putative, expressed; mechanosensitive channel of small conductance-like 6
PF00004	1	uncharacterized protein ycf45, putative, expressed; P-loop containing nucleoside triphosphate hydrolases superfamily protein

PF00719	1	soluble inorganic pyrophosphatase, putative, expressed; pyrophosphorylase 6
PF00642	1	zinc finger/CCCH transcription factor, putative, expressed; Zinc finger C-x8-C-x5-C-x3-H type family protein
PF07944	1	DUF1680 domain containing protein, putative, expressed; Putative glycosyl hydrolase of unknown function (DUF1680)
PF17123,PF13519	1	zinc finger family protein, putative, expressed; Zinc finger (C3HC4-type RING finger) family protein
PF01408	1	uncharacterized oxidoreductase, putative, expressed; Oxidoreductase family protein
PF05347	1	LYR motif containing protein, putative, expressed; LYR family of Fe/S cluster biogenesis protein
PF04727	1	ELMO/CED-12 family protein, putative, expressed; ELMO/CED-12 family protein
PF07887	1	calmodulin binding protein, putative, expressed; Calmodulin binding protein-like
PF00097	1	zinc finger, C3HC4 type domain containing protein, expressed; RING/U-box superfamily protein
PF00636	1	RNA binding protein, putative, expressed; Ribonuclease III family protein
PF01344,PF13418, PF13415	1	kelch repeat protein, putative, expressed; Galactose oxidase/kelch repeat superfamily protein
PF00107,PF16884	1	NADP-dependent oxidoreductase, putative, expressed; Zinc-binding dehydrogenase family protein
PF00641	1	zinc finger family protein, putative, expressed; Ran BP2/NZF zinc finger-like superfamily protein
PF00122,PF00689, PF00702	1	calcium-transporting ATPase, plasma membrane-type, putative, expressed; ATPase E1-E2 type family protein / haloacid dehalogenase-like hydrolase family protein
PF01734	1	phospholipase, patatin family, putative, expressed; Acyl transferase/acyl hydrolase/lysophospholipase superfamily protein
PF14008,PF00149, PF16656	1	nucleotide pyrophosphatase/phosphodiesterase, putative, expressed; purple acid phosphatase 27
PF00967,PF00187	1	WIP3 - Wound-induced protein precursor, expressed; pathogenesis-related 4
PF07983,PF00332	1	glucan endo-1,3-beta-glucosidase precursor, putative, expressed; O-Glycosyl hydrolases family 17 protein
PF09258	1	exostosin, putative, expressed; glycosyltransferase family protein 47
PF06280,PF02225, PF05922,PF00082	1	OsSub52 - Putative Subtilisin homologue, expressed; subtilisin-like serine protease 3
PF07732,PF07731, PF00394	1	laccase precursor protein, putative, expressed; laccase 2
PF05512	1	AWPM-19-like membrane family protein, putative, expressed; AWPM-19-like family protein
PF01925	1	membrane protein, putative, expressed; Sulfite exporter TauE/SafE family protein
PF07786	1	heparan-alpha-glucosaminide N-acetyltransferase, putative, expressed; Protein of unknown function (DUF1624)
PF01453,PF00069	1	lectin protein kinase family protein, putative, expressed; S-domain-2 5
PF03703	1	putativ eribonuclease P, putative, expressed; NAD(P)-linked oxidoreductase superfamily protein
PF03070	1	TENA/THI-4 family protein, putative, expressed; Haem oxygenase-like, multi-helical
PF12734	1	NA
PF05368	1	isoflavone reductase homolog IRL, putative, expressed; NmrA-like negative transcriptional regulator family protein
PF02141,PF03456	1	DENN domain containing protein, expressed; DENN (AEX-3) domain-containing protein
PF07491	1	NA

PF13292,PF02779, PF02780	1	transketolase, putative, expressed; Deoxyxylulose-5-phosphate synthase
PF06911	1	senescence-associated protein, putative, expressed; Senescence/dehydration-associated protein-related
PF01373	1	beta-amylase, putative, expressed; beta-amylase 3
PF13417,PF13410	1	glutathione S-transferase, N-terminal domain containing protein, expressed; dehydroascorbate reductase 2
PF02798,PF00043	1	glutathione S-transferase, putative, expressed; glutathione S-transferase TAU 8
PF05742	1	Ser/Thr-rich protein T10 in DGCR region, putative, expressed; Protein of unknown function (DUF833)
PF03283	1	pectinacetylesterase domain containing protein, expressed; Pectinacetylesterase family protein
PF07714,PF04564	1	protein kinase, putative, expressed; U-box domain-containing protein kinase family protein
PF00505,PF01388	1	high mobility group, putative, expressed; HMG (high mobility group) box protein with ARID/BRIGHT DNA-binding domain
PF02728,PF01179, PF02727	1	copper methylamine oxidase precursor, putative, expressed; Copper amine oxidase family protein
PF13439,PF00534	1	glycosyl transferase, group 1 domain containing protein, expressed; UDP-Glycosyltransferase superfamily protein
PF12215,PF04685	1	non-lysosomal glucosylceramidase, putative, expressed; Beta-glucosidase, GBA2 type family protein
PF00988,PF00117	1	class I glutamine amidotransferase, putative, expressed; carbamoyl phosphate synthetase A
PF02874,PF00306, PF00006	1	ATP synthase, putative, expressed; ATP synthase subunit 1
PF00560,PF00069, PF08263,PF13855	1	NA
PF13962	1	NA
PF05030	1	SSXT protein, putative, expressed; GRF1-interacting factor 3
PF06217	1	GAGA-binding protein, putative, expressed; basic pentacysteine 6
PF13249,PF13243	1	cycloartenol synthase, putative, expressed; Terpenoid cyclases family protein
PF00860	1	permease domain containing protein, putative, expressed; AZA-guanine resistant1
PF02353,PF01593	1	Cyclopropane-fatty-acyl-phospholipid synthase, putative, expressed; Cyclopropane-fatty-acyl-phospholipid synthase
PF00459	1	inositol-1-monophosphatase, putative, expressed; HAL2-like
PF03760	1	NA
PF00202	1	aminotransferase, putative, expressed; PYRIMIDINE 4
PF01486,PF00319	1	OsMADS47 - MADS-box family gene with MIKCc type-box, expressed; K-box region and MADS-box transcription factor family protein
PF00462	1	expressed protein; Glutaredoxin family protein
PF13460	1	dihydroflavonol-4-reductase, putative, expressed; NAD(P)-binding Rossmann-fold superfamily protein
PF00326	1	OsPOP12 - Putative Prolyl Oligopeptidase homologue, expressed; alpha/beta-Hydrolases superfamily protein
PF00170,PF14144	1	transcription factor, putative, expressed; bZIP transcription factor family protein
PF01073	1	sterol-4-alpha-carboxylate 3-dehydrogenase, decarboxylating, putative, expressed; 3-beta hydroxysteroid dehydrogenase/isomerase family protein

PF06888	1	phosphoethanolamine/phosphocholine phosphatase, putative, expressed; Pyridoxal phosphate phosphatase-related protein
PF00722,PF06955	1	glycosyl hydrolases family 16, putative, expressed; xyloglucan endotransglucosylase/hydrolase 6
PF14709,PF00636	1	NA
PF05153	1	inositol oxygenase, putative, expressed; myo-inositol oxygenase 4
PF06547,PF13639, PF14369	1	zinc finger, RING-type, putative, expressed; zinc finger (C3HC4-type RING finger) family protein
PF01764	1	lipase, putative, expressed; alpha/beta-Hydrolases superfamily protein
PF04367	1	protein of unknown function DUF502 domain containing protein, expressed; Protein of unknown function (DUF502)
PF01263	1	aldose 1-epimerase, putative, expressed; Galactose mutarotase-like superfamily protein
PF08501,PF01487	1	bifunctional 3-dehydroquinate dehydratase/shikimate dehydrogenase,chloroplast precursor, putative, expressed; dehydroquinate dehydratase, putative / shikimate dehydrogenase, putative
PF00215,PF00156	1	uridine 5-monophosphate synthase, putative, expressed; uridine 5\'-monophosphate synthase / UMP synthase (PYRE-F) (UMPS)
PF07172	1	NA
PF01758	1	bile acid sodium symporter family protein, putative, expressed; Sodium Bile acid symporter family
PF01565,PF09265	1	cytokinin dehydrogenase precursor, putative, expressed; cytokinin oxidase 7
PF00117	1	class I glutamine amidotransferase, putative, expressed; Glutamine amidotransferase type 1 family protein
PF04859	1	GIL1, putative, expressed; Plant protein of unknown function (DUF641)
PF03000,PF00651	1	NA
PF04525	1	DUF567 domain containing protein, putative, expressed; Protein of unknown function (DUF567)
PF13855,PF00560, PF00069,PF12799, PF08263	1	NA
PF05726,PF02678	1	pirin, putative, expressed; RmlC-like cupins superfamily protein
PF13537,PF00733	1	asparagine synthetase, putative, expressed; glutamine-dependent asparagine synthase 1
PF07690	1	transporter, major facilitator family, putative, expressed; Major facilitator superfamily protein
PF00415	1	Regulator of chromosome condensation domain containing protein, expressed; Regulator of chromosome condensation (RCC1) family protein
PF07714,PF11721, PF13855,PF00560	1	Leucine-rich repeat transmembrane protein kinase
PF06075	1	expressed protein; Plant protein of unknown function (DUF936)
PF12143,PF00264, PF12142	1	NA
PF01716	1	oxygen-evolving enhancer protein 1, chloroplast precursor, putative, expressed; photosystem II subunit O-2
PF07716,PF14144	1	transcription factor, putative, expressed; bZIP transcription factor family protein
PF04689,PF00069, PF08263,PF13855	1	inactive receptor kinase At2g26730 precursor, putative, expressed; Leucine-rich repeat protein kinase family protein

PF02892,PF03514, PF14372,PF02902, PF05699	1	NA
PF06521	1	PAR1 protein
PF13855,PF00560, PF08263	1	NA
PF04844,PF13724	1	NA
PF08263,PF13855	1	uncharacterized protein At4g06744 precursor, putative, expressed; Leucine-rich repeat (LRR) family protein
PF08030,PF01794, PF08414,PF08022	1	respiratory burst oxidase, putative, expressed; respiratory burst oxidase homologue D
PF03098	1	alpha-DOX2, putative, expressed; Peroxidase superfamily protein
PF01426,PF00628	1	ES43 protein, putative, expressed; PHD finger family protein / bromo-adjacent homology (BAH) domain-containing protein
PF03468,PF03469, PF03470	1	transcription factor X1, putative, expressed; XH/XS domain-containing protein
PF01388,PF00505	1	high mobility group, putative, expressed; HMG (high mobility group) box protein with ARID/BRIGHT DNA-binding domain
PF00076,PF12872, PF12796	1	NA
PF13813	1	wax synthase isoform 1, putative, expressed; acyl-CoA sterol acyl transferase 1
PF05199,PF00732	1	HOTHEAD precursor, putative, expressed; Glucose-methanol-choline (GMC) oxidoreductase family protein
PF03791,PF03790, PF03789,PF05920	1	Homeobox domain containing protein, expressed; KNOTTED-like from Arabidopsis thaliana
PF00042	1	non-symbiotic hemoglobin 2, putative, expressed; haemoglobin 2
PF10184,PF04832	1	SOUL heme-binding protein, putative, expressed; SOUL heme-binding family protein
PF00931	1	NA
PF06839	1	NA
PF05056	1	NA
PF03595	1	C4-dicarboxylate transporter/malic acid transport protein domain containing protein, expressed; SLAC1 homologue 1
PF02225,PF05922, PF00082	1	OsSub58 - Putative Subtilisin homologue, expressed; Subtilase family protein

Table 5. Gene expression matrix (Log TMM+1) of the 154 overlapping differentially expressed genes in Coker312 and Jin668.

gene_id	Coker312_ec	Coker312_nec	Jin668_ec	Jin668_nec	Gene Function	Best Hit Arabidopsis	Fold Change (Jin668 EC vs Coker312 EC)
Gohir.D07G080100.1	10.9739565	7.503865481	13.3577685	10.4382679	Bifunctional inhibitor/lipid-transfer protein/seed storage 2S albumin superfamily protein	NA	1.21722448
Gohir.A07G075600.1	3.74899785	0	11.6349308	8.69948223	Bifunctional inhibitor/lipid-transfer protein/seed storage 2S albumin superfamily protein	NA	3.10347759
Gohir.D13G121100.1	9.26393464	0.240924865	10.6401473	6.72679035	0	NA	1.14855596
Gohir.D13G121201.1	9.26393464	0.240924865	10.6401473	6.72679035	0	NA	1.14855596
Gohir.D13G121301.1	9.26393464	0.240924865	10.6401473	6.72679035	0	NA	1.14855596
Gohir.D13G121400.1	9.26393464	0.240924865	10.6401473	6.72679035	0	NA	1.14855596
Gohir.D13G121500.1	9.26393464	0.240924865	10.6401473	6.72679035	0	NA	1.14855596
Gohir.D13G121601.1	9.26393464	0.240924865	10.6401473	6.72679035	0	NA	1.14855596
Gohir.D13G121700.1	9.26393464	0.240924865	10.6401473	6.72679035	0	NA	1.14855596
Gohir.D07G195400.1	6.37046074	2.689690536	9.81671223	6.59292042	Glycine-rich protein family	NA	1.54097366
Gohir.A02G027300.1	6.80970061	1.297925053	9.70227487	6.00462124	lipid transfer protein 1	AT2G38540	1.4247726
Gohir.D02G178800.1	6.57924804	2.212102372	8.43335454	5.35033222	early nodulin-like protein 3	AT4G32490	1.28181131
Gohir.A07G043800.1	6.94758142	2.960187327	8.40070585	4.87735163	oleosin 1	AT4G25140	1.20915544
Gohir.D11G255800.1	6.45101324	0.705314441	8.29483974	4.9399631	homeobox protein 31	NA	1.28581967
Gohir.A09G247200.1	6.00399007	3.238863271	7.91180941	4.85263146	Heavy metal transport/detoxification superfamily protein	NA	1.31775858
Gohir.A03G155700.1	5.94891617	1.632035504	7.7557533	4.77638478	early nodulin-like protein 3	AT4G32490	1.30372543
Gohir.A09G213654.1	4.98415639	1.44903306	7.74337359	3.55691984	lipid transfer protein 6	AT3G08770	1.55359764
Gohir.A05G157800.2	6.22391965	1.181579659	7.70281682	3.72450476	homeobox-3	AT2G33880	1.23761508
Gohir.D06G172800.3	6.08489872	1.281549893	7.61528141	4.44019921	0	NA	1.25150504
Gohir.D09G247800.1	5.29303935	2.845991771	7.07594259	4.2131658	Heavy metal transport/detoxification superfamily protein	AT5G63530	1.33683922
Gohir.A13G117900.1	5.21310422	0	6.99139335	3.2890475	0	NA	1.34111904
Gohir.D01G170800.1	5.68572294	1.048759312	6.87953832	3.38809767	D-aminoacid aminotransferase-like PLP-dependent enzymes superfamily protein	AT1G50110	1.20996721
Gohir.D13G136000.1	4.98069941	0.638537279	6.7394881	3.10980628	Histone superfamily protein	AT1G21970	1.35312083
Gohir.D13G183400.1	4.88670865	1.270528942	6.68759731	3.20434942	nodulin MtN21 /EamA-like transporter family protein	AT1G09380	1.36852794
Gohir.D10G099700.1	4.47654371	1.265136803	6.68541693	3.50224572	AINTEGUMENTA-like 6	AT5G10510	1.49343274
Gohir.D05G281700.1	5.02861335	0.811676528	6.65367152	4.26233293	Peroxidase superfamily protein	AT5G05340	1.32316228

Gohir.A13G176900.1	5.02343279	1.114367025	6.60252319	3.17184731	nodulin MtN21 /EamA-like transporter family protein	AT1G09380	1.31434488
Gohir.D05G179300.1	5.04575966	0.743945752	6.56614032	2.95902822	nuclear factor Y, subunit B6	AT5G47670	1.30131848
Gohir.D05G160500.2	5.34595516	0.620820976	6.44393259	3.0141172	homeobox-3	AT2G33880	1.20538471
Gohir.D09G214700.1	5.30594317	0	6.28909055	2.57224294	lipid transfer protein 6	AT3G08770	1.18529173
Gohir.A05G176400.1	4.53791725	0.523561956	6.24466511	2.67144435	nuclear factor Y, subunit B6	AT5G47670	1.37610819
Gohir.D13G121800.1	6.12237765	0	6.22208324	2.95382395	NA	NA	1.01628544
Gohir.D11G128300.1	5.1947864	1.719402512	6.216623	3.77634968	Cupredoxin superfamily protein	AT5G26330	1.19670426
Gohir.A07G221900.1	4.07328469	1.809723119	6.20020222	3.31058249	myb domain protein 118	NA	1.52216275
Gohir.D11G269700.2	4.90632543	0.319906907	6.15607296	2.93237619	glutamate dehydrogenase 1	AT5G18170	1.2547217
Gohir.D10G116400.1	4.61075911	1.853097425	6.09691541	4.17080638	lipid transfer protein 1	AT2G38540	1.32232356
Gohir.D05G290600.1	2.1462479	4.525802665	6.08862219	3.82249223	Nucleotide-diphospho-sugar transferases superfamily protein	AT5G03760	2.83686809
Gohir.A09G175400.1	5.46918594	1.72704913	6.03336426	2.45663095	PEBP (phosphatidylethanolamine-binding protein) family protein	AT1G18100	1.10315581
Gohir.A10G197700.2	3.68818036	0.031395196	5.98425514	2.05716142	cytochrome P45, family 77, subfamily A, polypeptide 4	AT5G04660	1.62254949
Gohir.A13G044200.1	4.26160603	1.114200422	5.91924473	2.63691458	basic helix-loop-helix (bHLH) DNA-binding superfamily protein	AT3G19500	1.38897042
Gohir.D12G154900.1	4.6196331	2.518849829	5.8723445	3.62729554	high mobility group A	NA	1.27117119
Gohir.D07G224000.1	3.45061742	0.180784391	5.86454935	3.32399448	ABC transporter family protein	AT3G28345	1.69956522
Gohir.D07G061900.1	3.70057843	0.244582661	5.85148254	3.06223686	strictosidine synthase 3	NA	1.58123457
Gohir.A10G150100.1	3.48468597	0	5.75454014	2.71633248	lipid transfer protein 1	AT2G38540	1.65137984
Gohir.D10G204800.1	2.92086493	0.147957881	5.69597526	1.83025532	cytochrome P45, family 77, subfamily A, polypeptide 4	AT5G04660	1.95009882
Gohir.A11G248800.1	3.36632221	0.192509819	5.6923528	1.60928218	ATPase E1-E2 type family protein / haloacid dehalogenase-like hydrolase family protein	AT3G22910	1.69097087
Gohir.D09G044700.1	5.20974577	2.614179777	5.66380002	3.43050831	subtilase 1.3	AT5G51750	1.08715478
Gohir.D01G200400.1	4.21971064	1.710393192	5.62307672	2.44112931	GDSL-like Lipase/Acylhydrolase family protein	AT5G03820	1.33257401
Gohir.D10G210100.1	4.42270395	0.423309237	5.60586962	2.37388017	Papain family cysteine protease	AT3G54940	1.26752088
Gohir.A08G163500.1	9.16131331	12.29330898	5.59932771	8.21479508	expansin-like B1	AT4G17030	0.61119269
Gohir.A11G151200.1	2.42485492	0	5.54439265	1.17120683	NA	NA	2.28648428
Gohir.D11G279600.1	3.91738401	0.301880253	5.51796852	2.13783137	alpha/beta-Hydrolases superfamily protein	AT3G05600	1.40858504
Gohir.D10G189500.1	3.56620629	0	5.48283753	2.42304025	annexin 8	AT5G12380	1.53744262
Gohir.A10G176400.1	4.22083295	1.842878292	5.4656045	2.57539302	pyruvate dehydrogenase kinase	AT3G06483	1.29491135
Gohir.D06G216000.1	3.37312616	0	5.4436619	1.69198075	0	AT1G12064	1.61383288
Gohir.D12G225800.1	3.24800344	0.350497247	5.4300622	1.66827044	growth-regulating factor 2	AT2G22840	1.67181541
Gohir.D09G058500.1	4.72377697	0.740064059	5.39290125	2.08031656	seed gene 1	AT4G26740	1.14165027

Gohir.D05G077700.1	2.79071992	0.100641316	5.33718776	1.94110631	tetraspanin3	AT5G60220	1.91247704
Gohir.D10G116300.1	3.043257	0	5.33230216	2.45934418	lipid transfer protein 3	AT5G59320	1.75216952
Gohir.A11G259600.1	4.14218877	0.612824276	5.30850893	2.06384804	glutamate dehydrogenase 1	AT5G18170	1.28157098
Gohir.A03G172500.2	4.27956415	0.312955542	5.2819084	1.94772849	Bifunctional inhibitor/lipid-transfer protein/seed storage 2S albumin superfamily protein	AT5G64080	1.23421643
Gohir.D02G195700.1	3.88978989	0.483880254	5.2812655	1.98732087	Bifunctional inhibitor/lipid-transfer protein/seed storage 2S albumin superfamily protein	AT5G64080	1.35772513
Gohir.D02G113100.1	3.94300705	1.6617496	5.27504431	2.45750674	PHYTOCYSTATIN 2	NA	1.33782269
Gohir.A05G252200.1	4.14041057	0.125651102	5.21723072	2.1256511	GDSL-like Lipase/Acylhydrolase superfamily protein	AT1G71691	1.26007569
Gohir.D08G183300.1	8.21335579	11.64098598	5.18143389	8.077316	expansin-like B1	AT4G17030	0.63085467
Gohir.A11G070300.1	2.4765113	0	5.11791664	1.58111018	0	NA	2.06658319
Gohir.A13G056200.1	3.5527152	0.716112942	5.11367272	3.21676526	alpha/beta-Hydrolases superfamily protein	NA	1.43937029
Gohir.A09G235000.1	4.42810888	2.117445707	5.10813411	2.78457378	Haem oxygenase-like, multi-helical	AT3G16990	1.15357012
Gohir.A10G193800.1	3.7977912	0.411697404	5.10456032	1.95183643	Integrase-type DNA-binding superfamily protein	AT3G54320	1.34408661
Gohir.D03G016600.1	3.64148823	0.597411988	5.09375734	2.25005203	UDP-glucosyl transferase 72E1	AT3G50740	1.39881198
Gohir.A01G059700.1	3.54942275	1.117030053	5.03998644	2.07963411	UDP-Glycosyltransferase superfamily protein	AT4G27560	1.41994538
Gohir.A07G116400.1	3.63558028	0.706862041	5.02112434	2.17504549	RmlC-like cupins superfamily protein	AT2G28490	1.38110671
Gohir.A11G086500.1	3.69426734	0.845590409	5.01297086	2.31981059	0	NA	1.35695942
Gohir.D05G252700.1	5.02706556	0.153805336	4.99975953	1.65550449	sucrose-proton symporter 2	AT1G71880	0.9945682
Gohir.D01G218600.1	2.40633258	0	4.99846632	2.3193289	Basic-leucine zipper (bZIP) transcription factor family protein	NA	2.07721342
Gohir.A03G078600.1	4.61466568	1.508428653	4.98293275	1.40853072	beta-tonoplast intrinsic protein	AT1G17810	1.07980363
Gohir.D02G118600.1	4.28885685	1.20851726	4.80408799	1.54761434	beta-tonoplast intrinsic protein	AT1G17810	1.12013251
Gohir.A05G168100.1	2.99792463	0.635290054	4.78729315	1.91953078	thiazole biosynthetic enzyme, chloroplast (ARA6) (THI1) (THI4)	AT5G54770	1.59686908
Gohir.A11G089500.1	3.36730117	0.708407983	4.77699881	1.71002577	Major facilitator superfamily protein	AT4G00370	1.41864317
Gohir.D07G003100.2	3.20241772	0.610463845	4.76309283	1.7506065	pleiotropic drug resistance 1	AT3G30842	1.48734277
Gohir.D08G186400.1	3.31469653	0.928465078	4.73266718	2.23542072	Homeobox-leucine zipper protein 4 (HB-4) / HD-ZIP protein	AT4G16780	1.42778295
Gohir.A05G009300.1	2.41176519	0	4.66887553	1.12521025	Ran BP2/NZF zinc finger-like superfamily protein	AT3G15680	1.93587484
Gohir.D07G120800.1	3.80273606	0.525317212	4.64358686	1.97746311	RmlC-like cupins superfamily protein	AT2G28490	1.22111732
Gohir.A02G147800.1	3.133769	0.718745198	4.59647802	2.09051473	HAESA-like 2	NA	1.46675713
Gohir.A11G076600.1	3.30673616	0.551885103	4.58301756	1.12432814	homeobox-leucine zipper protein 3	AT3G60390	1.38596409
Gohir.D10G148500.1	3.02962957	0.65718266	4.5814316	1.98707827	Major facilitator superfamily protein	AT2G32040	1.5122085
Gohir.A09G127800.1	5.24308828	2.650190123	4.57171726	1.2654369	Late Embryogenesis Abundant 4-5	NA	0.87195123
Gohir.D07G129500.1	3.06854184	0.192194165	4.56642957	1.49518348	Integrase-type DNA-binding superfamily protein	AT1G51190	1.48814317
Gohir.D11G093600.1	4.39092558	0.300416628	4.55716498	0.71954854	plasma membrane intrinsic protein 1;4	AT1G01620	1.03785976

Gohir.D06G141500.1	4.80171482	0.946356572	4.55233608	2.06338789	chlorophyll A/B binding protein 1	AT1G29930	0.94806465
Gohir.A11G123000.1	3.68366839	0.656496371	4.54281521	1.99228503	Cupredoxin superfamily protein	AT5G26330	1.23323131
Gohir.D13G233400.1	2.89234245	0.031395196	4.5387037	1.46519089	WUSCHEL related homeobox 2	AT5G59340	1.5692138
Gohir.D13G173200.1	7.52529372	10.11524885	4.52969603	7.36330543	threonine aldolase 1	AT1G08630	0.60192947
Gohir.D13G110500.2	4.03703073	0.204453768	4.52884216	0.80074462	Glycoprotein membrane precursor GPI-anchored	AT1G54860	1.12182504
Gohir.D01G028100.2	3.45710175	0	4.51826235	1.44378344	Uncharacterised protein family (UPF497)	AT1G17200	1.30695093
Gohir.D01G046100.1	3.49156453	0.830863757	4.50269127	1.69346837	UDP-Glycosyltransferase superfamily protein	AT5G54010	1.28959131
Gohir.D05G122000.2	3.00786824	0.034919984	4.49942072	1.1514259	NA	NA	1.49588358
Gohir.A07G125100.1	3.03188037	0.086104371	4.48890057	1.26883359	Integrase-type DNA-binding superfamily protein	AT1G51190	1.48056652
Gohir.D05G254000.1	3.26153082	0.141759747	4.46830092	1.21805783	GDSL-like Lipase/Acylhydrolase superfamily protein	AT1G71691	1.37000114
Gohir.D13G218000.3	3.3281543	0.222186307	4.40689943	1.20309672	fatty acid desaturase 2	AT3G12120	1.32412714
Gohir.D09G094900.1	3.06522762	0.291014101	4.37469175	1.18014786	Basic-leucine zipper (bZIP) transcription factor family protein	NA	1.42719964
Gohir.D10G029500.1	2.6407366	0.18173866	4.36825617	1.3461532	homeobox-3	AT2G33880	1.65418095
Gohir.A05G079700.3	3.65966773	1.191562651	4.34186718	1.95729555	Pollen Ole e 1 allergen and extensin family protein	AT2G27385	1.18641022
Gohir.D13G115000.1	2.64898306	0.13060145	4.33885224	2.13608199	calreticulin 1a	AT1G56340	1.63793129
Gohir.A08G129000.1	3.84594161	0.215989154	4.29219277	1.14752379	vacuolar iron transporter 1	NA	1.11603171
Gohir.D11G092300.3	2.93663794	0.686164326	4.28813705	1.1256511	AT hook motif DNA-binding family protein	AT3G61310	1.46021986
Gohir.A13G167800.1	6.99416106	9.459028878	4.23314669	6.83778349	threonine aldolase 1	AT1G08630	0.60524009
Gohir.A13G214300.1	3.09621968	0.034919984	4.16280083	0.92447999	fatty acid desaturase 2	AT3G12120	1.34447851
Gohir.D04G179300.1	3.84714506	0.500292177	4.15445359	1.54234061	tryptophan aminotransferase of Arabidopsis 1	AT1G70560	1.07987963
Gohir.D04G037100.1	4.12536181	0.02467428	4.14963874	1.10277009	protodermal factor 1	NA	1.0058848
Gohir.D10G183100.1	2.83000173	0.155101558	4.14831048	1.00168216	pyruvate dehydrogenase kinase	AT3G06483	1.46583319
Gohir.A03G100700.1	3.17967028	0.530070742	4.10584567	1.3510629	NAC (No Apical Meristem) domain transcriptional regulator superfamily protein	AT1G26870	1.29128032
Gohir.A13G227800.1	2.9721412	0	4.07906516	1.30781708	WUSCHEL related homeobox 2	AT5G59340	1.37243317
Gohir.A10G201800.1	2.92333937	0.162532318	4.0646817	1.10321786	Papain family cysteine protease	AT3G54940	1.39042416
Gohir.A05G170500.2	3.03307054	0.668799909	4.02100586	1.45066141	Protein of unknown function, DUF584	AT4G21970	1.32572118
Gohir.A11G278000.2	3.22666266	0.809414444	3.97410097	0.96248733	F-box family protein	NA	1.23164439
Gohir.D08G035600.1	2.71550904	0.334854269	3.94828935	1.65443554	Histone superfamily protein	AT1G21970	1.45397761
Gohir.D09G123900.1	5.84670015	2.706309518	3.93913253	1.27361535	Late Embryogenesis Abundant 4-5	NA	0.67373603
Gohir.D01G063600.1	2.97181942	0	3.90926108	1.15574923	germin-like protein 5	NA	1.31544368
Gohir.D13G062100.1	2.74878323	0.131918679	3.88124043	1.17887396	glutamine synthase clone R1	AT5G37600	1.41198491
Gohir.A13G145600.2	3.99760856	1.046316431	3.85456091	1.17248752	AGAMOUS-like 8	NA	0.96421669
Gohir.D04G072300.1	2.60768539	0.222804561	3.71266914	1.25540002	Fatty acid hydroxylase superfamily	AT1G02205	1.4237412
Gohir.A10G212601.1	3.69857966	0	3.70800325	1.04986307	Protein of unknown function (DUF674)	NA	1.00254789
Gohir.A09G137100.1	4.06535689	0	3.70284221	1.24305971	0	AT5G06270	0.91082833

Gohir.A05G161100.1	5.50986208	7.819653815	3.66121753	8.1624484	arabinogalactan protein 3	NA	0.66448442
Gohir.D04G098600.1	2.91910168	0	3.6493472	0.71017275	0	AT3G52610	1.25016104
Gohir.A07G107300.1	3.06548615	0	3.61823866	0.36550591	PHYTOSULFOKINE 3 PRECURSOR	AT3G44735	1.1803148
Gohir.D02G125500.1	2.80560201	0	3.51075098	0.82971428	NAC (No Apical Meristem) domain transcriptional regulator superfamily protein	AT1G26870	1.25133606
Gohir.A03G107800.1	2.88811003	0	3.42729329	0.84022822	phytosulfokine 4 precursor	NA	1.18669069
Gohir.A05G214400.1	2.53642596	0	3.39812231	0.92194405	amino acid permease 6	AT5G49630	1.33972856
Gohir.A08G000012.1	0	3.172847504	3.23837926	0	NA	NA	3.23837926
Gohir.D05G217700.1	2.48812935	0.189982654	3.16858855	0.80157257	amino acid permease 6	AT5G49630	1.27348225
Gohir.A05G226700.1	2.52950864	0	2.99939875	0.44289927	0	AT1G78170	1.18576339
Gohir.A05G075600.1	3.18650056	0	2.99681053	0.6078629	0	NA	0.94047074
Gohir.A09G085000.1	3.18090371	0.586404475	2.96001738	0.07815437	Late embryogenesis abundant protein (LEA) family protein	NA	0.93055863
Gohir.A07G154900.2	3.71970368	0.189033824	2.9242266	0.37703118	AP2/B3-like transcriptional factor family protein	AT3G24650	0.78614504
Gohir.A03G054400.1	3.22009774	0.740495874	2.90297398	0.48645696	hydroxysteroid dehydrogenase 1	AT5G50600	0.90151735
Gohir.A05G378400.1	3.54151484	0	2.90059313	0.5620185	protodermal factor 1	NA	0.81902611
Gohir.D07G048700.2	6.82351055	9.161349846	2.82863158	5.1036515	NA	NA	0.41454198
Gohir.A09G028800.1	3.54831331	0.192194165	2.62269613	0.14099673	Oleosin family protein	AT3G01570	0.73913882
Gohir.D11G223400.1	4.1357811	0	2.61227385	0	heat shock factor binding protein	AT4G15802	0.63162769
Gohir.A08G163400.2	5.57979864	8.468388835	2.47836786	4.69519581	expansin-like B1	AT4G17030	0.44416797
Gohir.D01G153100.1	5.31874163	7.40921271	2.19870368	5.09224973	NA	NA	0.41338795
Gohir.A08G001700.1	4.98550043	2.964860664	2.14925937	4.22986987	copper transporter 1	AT2G26975	0.43110203
Gohir.D12G152400.2	3.80601465	5.969127461	1.7464561	3.99623808	Galactose oxidase/kelch repeat superfamily protein	AT1G67480	0.45886741
Gohir.A05G079900.2	3.83435727	6.563062771	1.51214259	4.52268353	0	NA	0.39436664
Gohir.D08G183200.1	5.26490231	8.213757964	1.50690655	3.74276079	expansin-like B1	AT4G17030	0.28621738
Gohir.A11G079800.1	4.09885324	6.967995683	1.30061186	3.74430464	Plant invertase/pectin methylesterase inhibitor superfamily	AT2G45220	0.31731116
Gohir.A09G072800.2	4.00854067	6.463029767	1.07701507	3.90204141	Protein of unknown function (DUF56)	AT4G32480	0.26868009
Gohir.D05G275600.2	5.12447289	9.163221332	0.9828263	3.66842174	NA	NA	0.19179071
Gohir.D05G156101.1	0	2.796026626	0	2.96156159	0	NA	0
Gohir.D08G188832.1	3.99451184	0	0	2.43571748	0	NA	0
Gohir.A11G265900.1	3.70055069	0.480006551	0	2.75730777	nuclear factor Y, subunit B5	AT2G47810	0
Gohir.D08G229750.1	5.0980742	0	0	5.74963259	Ribosomal protein L18ae/LX family protein	AT2G34480	0

Table 6. Functional enrichment categories of the 154 genes that are commonly differentially expressed among Coker312 and Jin668.

category	over_represented_pvalue	under_represented_pvalue	numDEInCat	numInCat	term	ontology	over_represented_FDR	go_term	gene_ids
GO:0006869	7.33E-11	1	8	93	lipid transport	BP	1.23E-07	BP lipid transport	Gohir.A02G027300.1, Gohir.D02G195700.1, Gohir.A10G150100.1, Gohir.D10G116300.1, Gohir.D10G116400.1, Gohir.D09G214700.1, Gohir.A07G075600.1, Gohir.D07G080100.1
GO:0009793	0.0001568	0.99999926	2	8	embryo development ending in seed dormancy	BP	0.0526535	BP embryo development ending in seed dormancy	Gohir.D09G123900.1, Gohir.A09G127800.1
GO:0008283	0.00206285	0.99995792	2	28	cell population proliferation	BP	0.3384943	BP cell population proliferation	Gohir.A03G107800.1, Gohir.A07G107300.1
GO:0006355	0.00221765	0.9994781	8	1069	regulation of transcription, DNA-templated	BP	0.3384943	BP regulation of transcription, DNA-templated	Gohir.D12G154900.1, Gohir.D02G125500.1, Gohir.A03G100700.1, Gohir.D09G094900.1, Gohir.D01G218600.1, Gohir.D12G225800.1, Gohir.D08G035600.1, Gohir.D13G136000.1
GO:0015770	0.01856991	0.99984476	1	10	sucrose transport	BP	1	BP sucrose transport	Gohir.D05G252700.1
GO:0009228	0.01926696	0.9998349	1	9	thiamine biosynthetic process	BP	1	BP thiamine biosynthetic process	Gohir.A05G168100.1
GO:0035434	0.04207551	0.99915679	1	18	copper ion transmembrane transport	BP	1	BP copper ion transmembrane transport	Gohir.A08G001700.1
GO:0005576	2.29E-05	0.99999882	5	142	extracellular region	CC	0.01280115	CC extracellular region	Gohir.D08G183300.1, Gohir.D08G183200.1, Gohir.A08G163500.1,

GO:0012511	0.00126317	0.99998023	2	22	monolayer-surrounded lipid storage body	CC	0.30298132	CC monolayer-surrounded lipid storage body	Gohir.A03G107800.1, Gohir.A07G107300.1<br><br>Gohir.A07G043800.1, Gohir.A09G028800.1
GO:0016021	0.00285237	0.99922946	9	1474	integral component of membrane	CC	0.39909361	CC integral component of membrane	Gohir.A08G001700.1, Gohir.D07G224000.1, Gohir.A07G043800.1, Gohir.A09G028800.1, Gohir.D13G183400.1, Gohir.A13G176900.1, Gohir.D05G077700.1, Gohir.D10G148500.1, Gohir.A11G089500.1
GO:0000785	0.01885107	0.99984443	1	8	chromatin	CC	1	CC chromatin	Gohir.D12G154900.1
GO:0005887	0.02316128	0.99975365	1	12	integral component of plasma membrane	CC	1	CC integral component of plasma membrane	Gohir.D05G252700.1
GO:0005634	0.04035814	0.99037494	4	641	nucleus	CC	1	CC nucleus	Gohir.D12G154900.1, Gohir.D12G225800.1, Gohir.D08G035600.1, Gohir.D13G136000.1
GO:0016020	0.04728387	0.9838781	6	1380	membrane	CC	1	CC membrane	Gohir.D06G141500.1, Gohir.D02G118600.1, Gohir.A03G078600.1, Gohir.D11G093600.1, Gohir.D13G183400.1, Gohir.A13G176900.1
GO:0008289	3.13E-10	1	8	129	lipid binding	MF	2.63E-07	MF lipid binding	Gohir.A02G027300.1, Gohir.D02G195700.1, Gohir.A10G150100.1, Gohir.D10G116300.1, Gohir.D10G116400.1, Gohir.D09G214700.1, Gohir.A07G075600.1, Gohir.D07G080100.1

GO:0046982	0.00011676	0.99999752	3	41	protein heterodimerization activity	MF	0.04900974	MF protein heterodimerization activity	Gohir.A11G265900.1, Gohir.D05G179300.1, Gohir.A05G176400.1
GO:0045735	0.00066405	0.99999255	2	20	nutrient reservoir activity	MF	0.18582259	MF nutrient reservoir activity	Gohir.A07G116400.1, Gohir.D07G120800.1
GO:0015267	0.00158877	0.99991478	3	98	channel activity	MF	0.33344208	MF channel activity	Gohir.D02G118600.1, Gohir.A03G078600.1, Gohir.D11G093600.1
GO:0003677	0.00204255	0.99931693	13	2462	DNA binding	MF	0.3384943	MF DNA binding	Gohir.D12G154900.1, Gohir.A11G076600.1, Gohir.A10G193800.1, Gohir.D10G029500.1, Gohir.D10G099700.1, Gohir.D08G186400.1, Gohir.A07G221900.1, Gohir.A07G125100.1, Gohir.D07G129500.1, Gohir.D13G233400.1, Gohir.A13G227800.1, Gohir.D02G125500.1, Gohir.A03G100700.1
GO:0008083	0.00339347	0.99990964	2	36	growth factor activity	MF	0.43717509	MF growth factor activity	Gohir.A03G107800.1, Gohir.A07G107300.1
GO:0009055	0.0036453	0.99957201	4	269	electron transfer activity	MF	0.43717509	MF electron transfer activity	Gohir.D11G128300.1, Gohir.A11G123000.1, Gohir.D02G178800.1, Gohir.A03G155700.1
GO:0016788	0.00777017	0.99926112	3	184	hydrolase activity, acting on ester bonds	MF	0.8697412	MF hydrolase activity, acting on ester bonds	Gohir.D05G254000.1, Gohir.A05G252200.1, Gohir.D01G200400.1
GO:0008234	0.01251301	0.99933136	2	79	cysteine-type peptidase activity	MF	1	MF cysteine-type peptidase activity	Gohir.A10G201800.1, Gohir.D10G210100.1
GO:0016758	0.01428433	0.99829837	3	252	transferase activity, transferring hexosyl groups	MF	1	MF transferase activity, transferring hexosyl groups	Gohir.D01G046100.1, Gohir.A01G059700.1, Gohir.D03G016600.1

GO:0008515	0.01856991	0.99984476	1	10	sucrose transmembrane transporter activity	MF	1	MF sucrose transmembrane transporter activity	Gohir.D05G252700.1
GO:0003714	0.02132025	0.99979516	1	10	transcription corepressor activity	MF	1	MF transcription corepressor activity	Gohir.D11G223400.1
GO:0003824	0.03167794	0.98909655	7	1598	catalytic activity	MF	1	MF catalytic activity	Gohir.D04G179300.1, Gohir.D13G173200.1, Gohir.D13G062100.1, Gohir.A13G167800.1, Gohir.A13G056200.1, Gohir.D11G279600.1, Gohir.D01G170800.1
GO:0005375	0.04207551	0.99915679	1	18	copper ion transmembrane transporter activity	MF	1	MF copper ion transmembrane transporter activity	Gohir.A08G001700.1

Table 7. List of primers used for RT-qPCR of embryogenesis genes.

Gene Name	Forward primer	Reverse primer	Acc. Num.	Amplicon size (bp)
GhLEC1	GAATGCGTCTCGGAGTACATAA	TCAACGTAGTCGTCGAAACC	Gohir.D13G136000.1	134
GhBBM	AATGGTGATGGGTGGTAGTTC	TATTGGCCGGTGGTTGTATC	Gohir.D08G247400.1	105
GhWOX5	GATGGAACCCTACGACTGAAC	GCTGTGTAGAAATCTTCTGGATTTG	Gohir.D10G245300.1	102
GhWUS	CCATGCAAACACCCATTCTTG	CCAGTTGAAGGAGGAGATGAAG	Gohir.D10G089500.1	115
